# Species‐habitat networks reveal key habitats for landscape‐level wild bee conservation

**DOI:** 10.1002/eap.70224

**Published:** 2026-04-01

**Authors:** Marit Kinga Kasten, Sara Tassoni, Thomas Hiller, Markus Röhl, Michael Roth, Ingo Grass

**Affiliations:** ^1^ Ecology of Tropical Agricultural Systems University of Hohenheim Stuttgart Germany; ^2^ Center for Biodiversity and Integrative Taxonomy (KomBioTa) University of Hohenheim Stuttgart Germany; ^3^ Institute for Landscape and Environment Nürtingen‐Geislingen University Nürtingen Germany

**Keywords:** agricultural intensification, farmland, insect decline, land use, landscape composition, meadow orchard

## Abstract

Most agricultural landscapes are composed of a variety of habitats. A landscape perspective is needed to understand biodiversity decline, but many studies focus on single habitat types. In addition, the use of local resources by species within and across habitats implies that species and their habitats are linked in species‐habitat networks. However, studies on these networks are scarce. Here, we used grid‐based sampling to assess wild bees at 224 sampling locations across all major habitat types, that is, arable land, grassland, forest and orchard, in 14 differently composed agricultural landscapes of Southern Germany. We assigned wild bees to habitat types based on the dominant habitat cover surrounding their sampling location to establish species‐habitat networks and assessed how these networks differed in modularity and robustness to habitat loss. Orchards harbored more wild bees than expected based on their proportional cover in the landscape, indicating a preference for this extensively managed but threatened habitat by wild bees. Orchards also supported the highest species richness and proportion of oligolectic wild bees, while forests harbored the lowest richness and more social species. Landscape diversity affected both structure and robustness of bee‐habitat networks in response to the simulated loss of habitats. Networks in more diverse landscapes had higher modularity but tended to be less robust, showing that greater landscape diversity and modularity do not necessarily buffer against the effects of habitat loss. However, this effect appeared to be mainly driven by increases in network size, as standardized modularity and robustness (z‐scores) were not affected by landscape diversity. We could show that species‐habitat networks are a powerful tool to inform ecologists and policy makers about the importance of key habitats and landscape diversity for species conservation. Key habitats for wild bee conservation include extensively managed habitats like traditional orchards. Nevertheless, all habitat types support a similar proportion of endangered species, emphasizing the importance of a diverse landscape. Conserving wild bees requires a variety of complementary habitats at the landscape scale and must consider the management of traditional and intensively managed habitats alike. Policy measures targeting landscape diversity are urgently needed.

## INTRODUCTION

Wild bees are among the most important pollinators globally, playing a vital role in both natural ecosystems and agricultural production (IPBES, 2016; Klein et al., 2007). However, in agricultural landscapes, wild bees are threatened by habitat loss and intensive agricultural practices (Potts et al., 2016). In Germany, half of the 560 wild bee species are on the red list of threatened species (Westrich et al., 2011), despite all being legally protected under the German Nature conservation law (§44). Ongoing declines in bee diversity indicate that past conservation efforts have failed to halt or reverse these declines.

To design effective conservation strategies against the decline of bees, we need to understand how they use resources in different habitats in heterogeneous landscapes (Marini et al., 2019). However, most studies on bee species and their conservation tend to focus on selected, discrete habitats embedded in such landscapes, such as calcareous grasslands (e.g., Jauker et al., 2013; Klaus et al., 2021) or arable land (e.g., Boff et al., 2024; Riedinger et al., 2015), while only few studies contain comparisons across habitat types (Ammann et al., 2024; Maurer et al., 2022; Scherber et al., 2019). Rather than focusing on a single or a few habitats, accurate assessment of landscape‐level drivers for bee conservation requires sampling methods that include all major habitat types within a study area. Grid‐based designs, where sampling intensity aligns with the proportional coverage of each habitat, are particularly suited to assess species diversity across heterogeneous landscapes (Scherber et al., 2019, 2021). Additionally, this study design enabled us to disentangle the influences of habitat types at the local scale and of habitat heterogeneity at the landscape scale (i.e., landscape diversity).

Recently, species‐habitat networks have been proposed as a novel tool for explicitly linking species and habitat resources in heterogeneous agricultural landscapes (Marini et al., 2019). Species‐habitat networks can assess the importance of specific habitats for species conservation in a given landscape and quantify emergent properties of entire habitat networks at the landscape level (Astudillo et al., 2020; Lami et al., 2021; Nardi et al., 2019). These properties include network modularity and robustness (Marini et al., 2019). For example, the modularity of species‐habitat networks can show how habitat types contribute to species communities at the landscape scale, and how this changes in more diverse landscapes with a greater number of habitat types (Marini et al., 2019). Species‐habitat networks with a more modular structure may therefore be at lower risk of collapse from the loss of specific habitats (Gilarranz et al., 2017; Martins et al., 2024). Yet, there are no empirical studies to date that evaluate species‐habitat networks and how their modularity relates to their robustness in differently diverse landscapes. In addition, simulations of habitat loss can provide information on the consequences of landscape homogenization and the loss of different habitats for biodiversity.

Here, we apply the species‐habitat network approach to study how habitat complementarity and landscape diversity affect wild bee diversity in agricultural landscapes. We use the tool to assess the relative importance of habitats for local bee diversity and the importance of landscape diversity (i.e., compositional diversity of habitats) for bee conservation in agricultural landscapes of Southern Germany. Major habitat types in these landscapes include extensively and intensively managed habitat types, that is, arable land, forests, grasslands, and orchards (Statistisches Landesamt Baden‐Württemberg, 2024b). Traditional habitats, such as extensively managed orchards, are endangered in Baden‐Württemberg according to the Red List of Biotope Types in Baden‐Württemberg (Breunig et al., 2020), while intensively farmed habitats like arable land are expanding in area. Consequently, traditional habitat types may be the first to disappear from these landscapes, while intensively managed habitat types are likely to persist longer. This may have consequences for bee diversity and the modularity and robustness of bee‐habitat networks in landscapes of differing habitat diversity (i.e., change with landscape diversity). We study bee‐habitat networks to infer key habitats and the consequences of landscape homogenization through habitat loss for bee assemblages.

## MATERIALS AND METHODS

### Landscape selection and mapping

**FIGURE 1:**
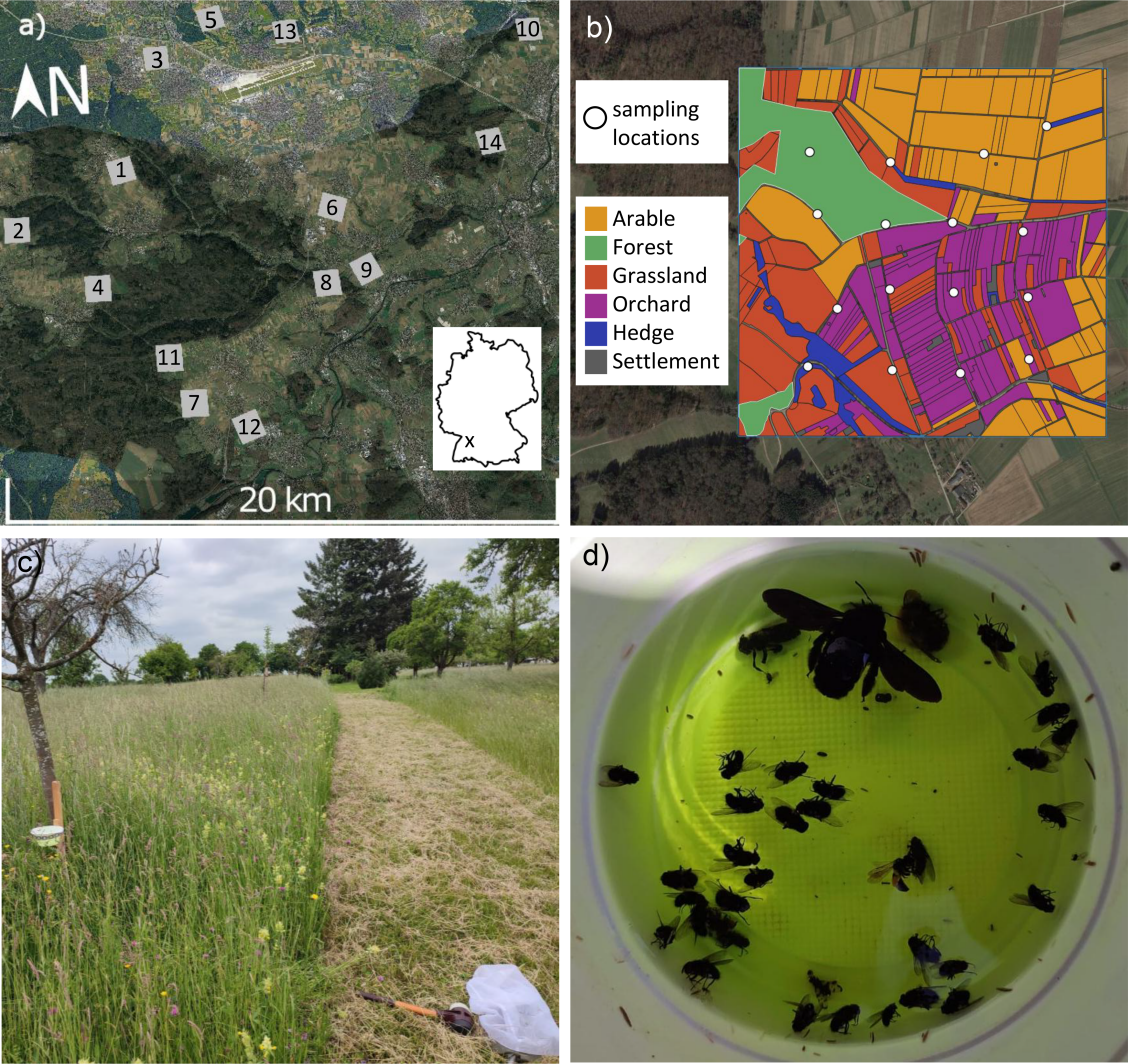
Overview of the grid‐based study design. (a) Distribution of the 14 study landscapes south of Stuttgart, Germany. The numbers in the squares identify the landscape ID; (b) grid‐based sampling design (Scherber et al., 2019) covering all habitat types (colored polygons) in each study landscape in proportion to their relative cover (example for study landscape L7) with white dots depicting the 16 sampling locations; (c) a sampling location with the sweep net and installed pan trap; (d) picture of a pan trap after 48 h sampling period. Maps were created by Marit K. Kasten and Sara Tassoni using QGIS version 3.36.2 with data from OpenStreetMap (https://www.openstreetmap.org/copyright) and land use information based on the IACS data (Metainformationssystem GDI‐BW, 2023). Image credits: Marit K. Kasten (panel c) and Carlos Gonzalez (panel d).

**FIGURE 2:**
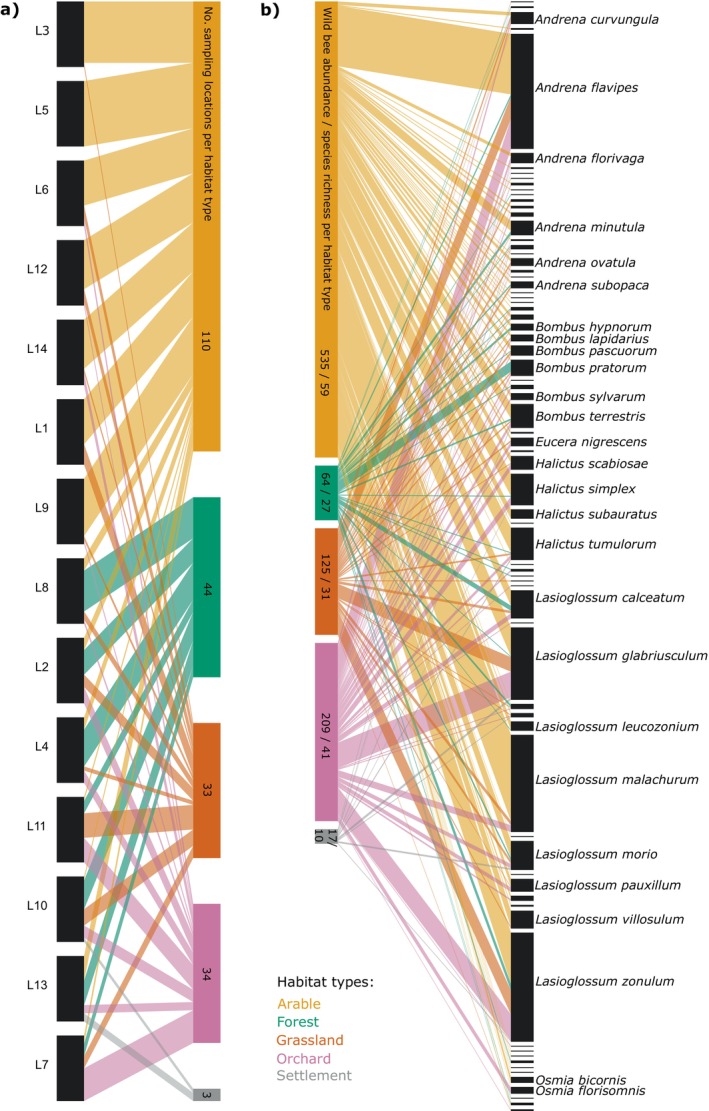
Metanetworks for habitat types per landscape and species per habitat type. (a) Habitat‐landscape metanetwork showing distribution of sampling locations across the different habitat types for the 14 landscapes; (b) species‐habitat metanetwork showing the species distribution over the habitat types across all 14 landscapes together. The landscapes (from top to bottom) are sorted by increasing landscape diversity; habitat types and bee species names are sorted alphabetically. For a full list of bee species names, see the Data sheet on bee species‐habitat metanetworks in Dryad. The colors of the links correspond to the color of the associated habitat types (arable: yellow, forest: green, grassland: brown, orchard: purple, settlement: gray). Values given in the boxes for the habitat types indicate in (a) the number of sampling locations assigned to each of the habitat types and in (b) the total wild bee abundance and species richness per habitat type across all landscapes together.

We conducted our study in an agricultural region (20 × 20 km^2^) south of Stuttgart, Germany (48°46′39″ N 09°10′48″ E). The landscapes in our study region mainly consist of arable land (dominated by cereals), forests (mainly dominated by beech), grasslands (including meadows and pastures), and orchards (mainly consisting of traditional, extensively managed apple orchards) (Appendix S1: Figure S1) (Streuobstportal Baden‐Württemberg, 2025; Statistisches Landesamt Baden‐Württemberg, 2024a; personal observation). In spring 2023, we identified 14 study landscapes of 1 × 1 km^2^ each with the aim to cover a gradient of landscape diversity from diverse landscapes with a balanced proportion of arable land, grassland, forest, and orchard to landscapes consisting predominantly of arable land (Figures 1a and 2a). Our study landscapes comprised the regional differences in land cover, land use, and surrounding habitats and were thus representative of Western European agricultural landscapes. The mean pairwise distance between the centers of the landscapes was 10.5 km ± 4.7 km (mean ± sd).

The classification of habitat types as grassland (defined as permanent grassland >5 years) or arable land was based on the IACS data (database of agricultural activities at field level; Metainformationssystem GDI‐BW, 2023) of the state of Baden‐Württemberg. Traditional orchards were classified on the basis of the state‐wide orchard survey (Borngraeber et al., 2020) and made up the majority of the orchards. Traditional orchards are characterized by sparsely arranged, often older standard trees with high stems, fully developed tree crowns and a grassland undergrowth that is often extensively grazed or mown. In contrast, intensively farmed orchards (potential pesticide use, high tree density arranged in rows, frequently mown undergrowth) were only present in one landscape. Other habitat types, including forests and settlements were mapped manually using QGIS v.3.36 (Figure 1b). Our study region is very densely populated; thus, there were many settlements. However, we tried to avoid these in our landscape selection so that they only made up a small proportion of our study landscapes (mean ± sd: 2.5% ± 2%). The remaining unmapped areas refer to roads, which can be grassy roads between fields or proper streets, whose cover was calculated. The GIS‐based habitat classifications were subsequently verified during fieldwork, during which we also mapped hedgerows in the study landscapes. We defined hedgerows as coherent, linear woody elements composed of shrubs and small trees, being only a few meters wide at maximum. We calculated landscape diversity as the Shannon diversity index for all seven habitat types (farmland, woodland, grassland, hedgerow, orchard, road, settlement) using their proportional cover as weights (Appendix S1: Figure S2 and see the Data sheet on Proportional cover of land cover types in landscapes in Dryad).

### Bee sampling and identification

We applied a grid‐based sampling design (Scherber et al., 2019), which allowed us to sample representatively across all major habitats, with the number of sampling locations proportional to their cover in the landscapes. To this end, we established 16 sampling locations in each study landscape (224 in total) with around 200 m distance between neighboring sampling locations whenever possible (Figure 1b). To sample wild bees, we used yellow pan traps and sweep netting at each of the 224 locations (Grundel et al., 2011; Kwaiser & Hendrix, 2008; Scherber et al., 2014; Westphal et al., 2008) (Figure 1c,d), because large bee species (e.g., bumblebees) are more difficult to sample with pan traps than smaller solitary bees. The sampling was repeated twice (first sampling period: 17 May—14 June 2023, second sampling period: 03 July—04 August 2023). Immediately before pan trap setup, we sweep‐netted wild bees for 3 min covering the vegetation in a 10‐m radius around each pan trap location. Sweep‐netted wild bees were killed in the field using ethyl acetate and subsequently stored in the freezer. Pan traps had a diameter of 14.3 cm and were painted with UV‐bright yellow color (Waldstein, neon yellow), put on poles of ca. 80 cm height, and filled with 300 mL of water and a drop of detergent to reduce water surface tension. Pan traps were left in the field for 48 h, and afterward samples were stored in 80% ethanol. Finally, insect samples were brought to the lab for bee identification. All bees sampled with both methods in both sampling rounds were pooled for each sampling location. Before sampling, permission to capture and collect wild bees was obtained from the regional council in Stuttgart (RPS55‐8850‐117/204/29).

From 1268 bees that we caught in total, we excluded 318 western honeybees (*Apis mellifera* L.) from all analyses, as their occurrence in our study region is mainly driven by beekeeping (Kohl et al., 2022). Over 99% of all remaining wild bee specimens were identified to species level by an expert taxonomist. We assessed sampling completeness of all 14 landscapes using species accumulation and extrapolation curves (R package iNEXT). Sampling completeness was calculated as the ratio of observed bee species richness to the estimated asymptotic richness (Chao, 1984) and correlated with landscape diversity.

### Bee‐habitat networks

In our analysis, we used species‐habitat networks, as proposed by Marini et al. (2019). We set up a bee species‐habitat network for each study landscape (Appendix S1: Figure S4). We assigned the sampled bees to habitats based on the habitat with the dominant proportional cover in a 100‐m radius around their sampling location (see the Data sheet on Dominant habitat type of sampling locations in Dryad). The number of locations assigned to their dominant habitat type closely matched its proportional cover in our study landscapes. This indicates that our sampling design and habitat assignment reflected the amounts of each habitat type across the landscapes, without any systematic under‐ or overrepresentation (Appendix S1: Figure S3). The interaction matrix underlying a particular bee‐habitat network consisted of bee species in columns and habitat types of the landscape in rows. We aggregated the sampled bees across all sampling locations of the same habitat type in a landscape to obtain matrix fills. Based on these interaction matrices, we assessed network size (i.e., the number of bee species multiplied by the number of habitat types per network) and correlated it with landscape diversity.

Additional to the sampling completeness of bee species, we also evaluated the sampling completeness of bee‐habitat interactions following Grass et al. (2018) and correlated it with landscape diversity. The calculation of this interaction completeness was based on the interaction matrix of each landscape. Interaction completeness was estimated using the Chao1 asymptotic richness estimator (Chao, 1984) which was calculated by dividing the observed interaction richness (*S*_obs_) by the estimated interaction richness. The estimated interaction richness was calculated as *S*_chao1_ = *S*_obs_ + (*F*_1_ × (*F*_1_ − 1))/(2 × (*F*_2_ + 1)), where *F*_1_ is the number of single interactions and *F*_2_ the number of double interactions.

We also set up a bee species‐habitat metanetwork in which all bee species and habitats were aggregated across the 14 study landscapes, and a habitat‐landscape metanetwork that indicates the number of sampling locations associated with a specific habitat type for all 14 landscapes (Figure 2).

### Statistical analysis

#### Habitat preference and avoidance of bees

Based on our networks generated in the previous step, we assessed the habitat preference or avoidance of wild bees. We wanted to know whether there are more or fewer bees in some habitats than would be expected from an even distribution of bees across the landscape's area. Habitat types can be used by wild bees for nesting and/or for foraging. As we used pan traps to attract foraging bees and sweep‐netted wild bees while foraging, preference, and avoidance are primarily based on the habitats' attractiveness for wild bees to forage there. Due to the small sample size of only three sampling locations assigned to the habitat type “settlement,” we removed these three sampling locations from all analyses.

**FIGURE 3:**
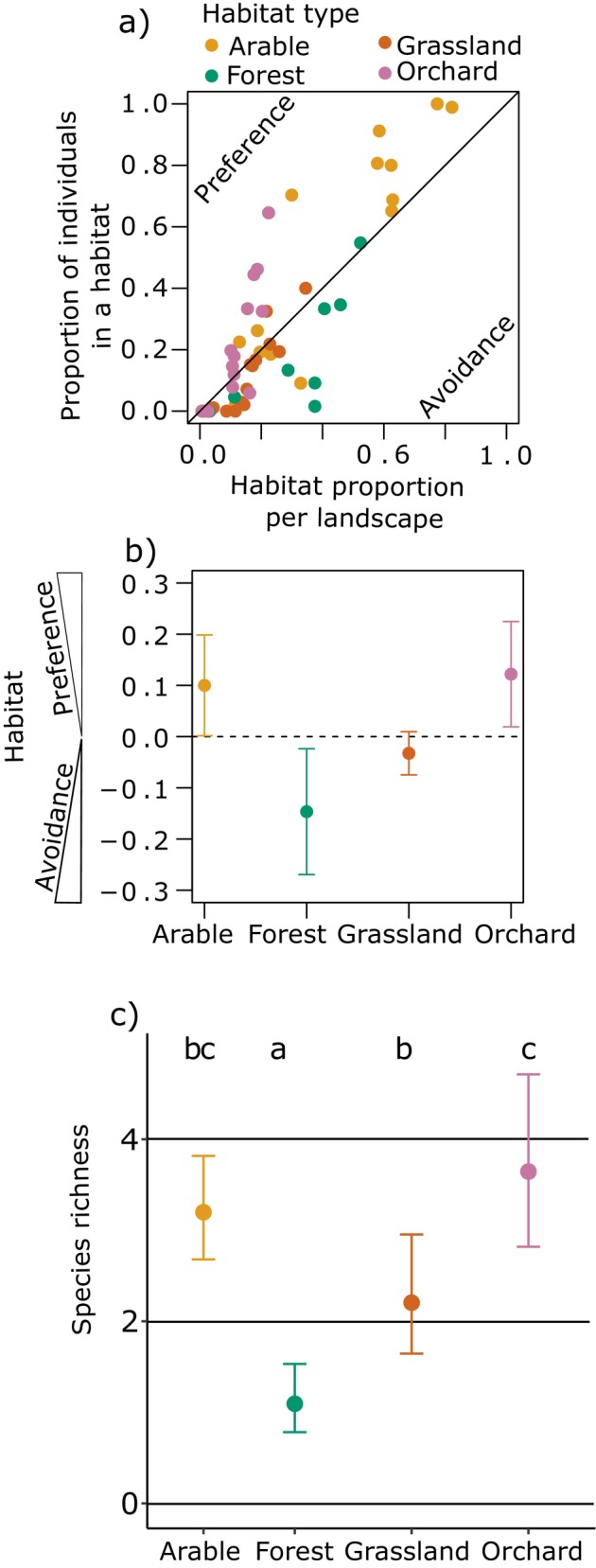
Habitat preference or avoidance of wild bees and species richness for the four focal habitat types. (a) For each landscape‐habitat combination, proportions of bees within each habitat type in the respective landscape's species‐habitat networks are shown compared to the habitat's actual proportion in the landscape. The diagonal line represents a perfect match between the relative proportion of bees within a habitat and the relative proportion of the habitat in the entire landscape (i.e., no preference or avoidance). The top left side indicates a wild bee's preference for the habitat in the respective landscape, the lower right side indicates an avoidance (for more detailed explanations, see the Methods). (b) Boxplots indicating the preference/avoidance of wild bees (mean and 95% CI) for the focal habitat types, summarized for all landscapes based on single dots from panel (a). (c) Species richness (mean and 95% CI) per habitat type. Different letters stand for significant differences between the habitat types.

We calculated the habitat preference or avoidance of bees by comparing their proportional occurrence in each habitat to their expected occurrence based on that habitat's proportional cover in the respective study landscape. Proportional occurrence refers to the number of individuals that were caught in a landscape's habitat (e.g., in L1's arable land or L7's orchard) proportional to the landscape's overall bee abundance. For example, if 25 bee individuals are caught in habitat ‘arable’ out of a total of 50 individuals in a landscape, the proportional occurrence is 0.5. In the absence of habitat preference or avoidance, the relative proportion of bees (e.g., 0.5) within a habitat would correspond to the relative proportion of the habitat in the respective landscape (e.g., arable land would make up 50% of the landscape's area; points would lie on the diagonal line in Figure 3a). Deviations from this expectation (i.e., the deviations from the diagonal in Figure 3a) indicate preference (higher bee density despite a lower proportional cover of the habitat) or avoidance (lower bee densities despite a large proportional cover). Finally, we aggregated all these deviations of all 14 landscapes for each habitat type (e.g., all yellow dots from Figure 3a for arable land; represented as mean and 95% confidence interval (CI) in Figure 3b). If the CI overlaps with the dashed line at 0 in Figure 3b, this means that wild bees neither preferred nor avoided this habitat across all landscapes. If the CI keeps above the 0, this indicates a habitat preference, below a habitat avoidance.

However, assigning a sampling location to a single habitat type may be arbitrary in the case of occurrence of multiple habitat types with similar shares in the 100 m radius. For this preference analysis, we additionally created probabilistic species‐habitat networks wherein habitat types of locations were assigned based on probabilities derived from the proportional covers (Appendix S1: Figure S5). For example, a sampling location with 30% arable land and 70% forest in the 100‐m radius would be assigned 300 times to arable land and 700 times to forest when creating 1000 probability‐assigned networks. To this end, we calculated the averages of the deviations over the 1000 replications for each landscape‐habitat combination and then again aggregated these deviations of all 14 landscapes for each habitat type.

#### Habitat and landscape effects on local species richness and functional composition of bees

We also wanted to understand if bee species richness and their functional traits were mainly driven by the identity of the local habitat type or rather by habitat heterogeneity at the landscape scale. Local habitat type and landscape diversity might also have interactive effects (e.g., arable land might have a higher species richness in complex landscapes than in simpler ones). Furthermore, a higher landscape‐wide cover of a certain local habitat type might also positively influence the species richness in that habitat type due to species‐area relationships acting on the regional species pool. Thus, we calculated the species richness of wild bees for each sampling location, which was assigned a dominant habitat type, a landscape diversity value, and the proportion of the respective habitat type in the respective landscape. To assess the influence of each of these variables on bee richness, we used generalized linear mixed‐effects models (glmmTMB with a negative binomial family to account for overdispersion). Fixed effects were the habitat type, landscape diversity, and their interaction as well as the habitat's proportion in the respective landscape. We included a random effect of landscape ID to account for nonindependence of sampling locations within the same landscape. Using stepwise backward model selection, we reduced the model until only statistically significant variables (*p* < 0.050) remained. With a post hoc Tukey test, we finally assessed differences among the habitat types. At the landscape scale, we also assessed the species richness and their correlation with landscape diversity (Pearson's correlation).

**FIGURE 4:**
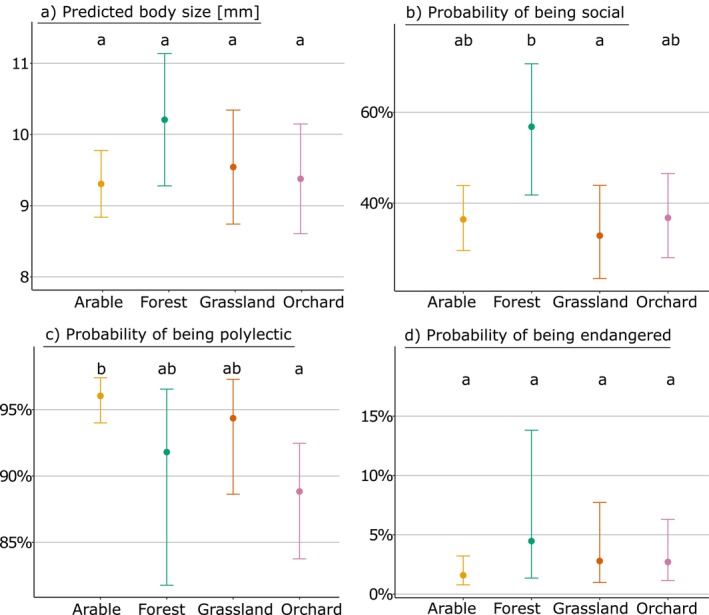
Traits of bee species per habitat types: (a) predicted body size [mm], (b) the probability of a bee being social instead of solitary, (c) the probability of a bee being polylectic instead of oligolectic, and (d) the probability of a bee being endangered (= IUCN red list category “endangered” or vulnerable”). Different letters stand for significant differences between the habitat types for each trait.

In addition to taxonomic variation, we also investigated whether functional traits mediate bee responses to habitats and landscape diversity. Traits included mean body size of female bees (mm; from literature and own measurements), sociality (solitary / social), dietary breadth (narrow = “oligolectic”/broad = “polylectic”), and endangerment status (not endangered/endangered = IUCN Category on German Red List endangered or vulnerable) (Scheuchl & Willner, 2024; Schwenninger et al., 2025; Westrich, 2019) (Figure 4). Bee species for which traits could not be retrieved were excluded from the respective trait analysis (excluding between 1 and 12 species, depending on the analysis; see the Data sheet on Trait information of bee species in Dryad).

For body size, we calculated the community weighted means for each sampling location and applied a linear mixed‐effects model. For each binomial trait, we counted the number of individuals for each characteristic in order to apply a generalized linear mixed‐effects model with binomial family. All the full mixed‐effects models included fixed effects of habitat type, landscape diversity, the habitat's proportion in the respective landscape and a random effect of landscape ID. An interaction term between habitat type and landscape diversity was only included for the body size, sociality, and dietary breadth traits, as it caused issues with model convergence for the endangerment status trait. We reduced these global models using stepwise backward model selection using ANOVA with Chi‐squared tests until only statistically significant terms remained.

#### Modularity of bee‐habitat networks

High network modularity means a network is subdivided into modules with dense interactions (here: between habitats and bee species) within modules and sparse connections between modules, which implies habitat niche differentiation within the ecological community (Dormann et al., 2009). Consequently, if bee species have highly differentiated niches and prefer different habitats, then modularity in diverse landscapes would be higher. Here, we wanted to know if the modularity of a network was influenced by landscape diversity.

**FIGURE 5:**
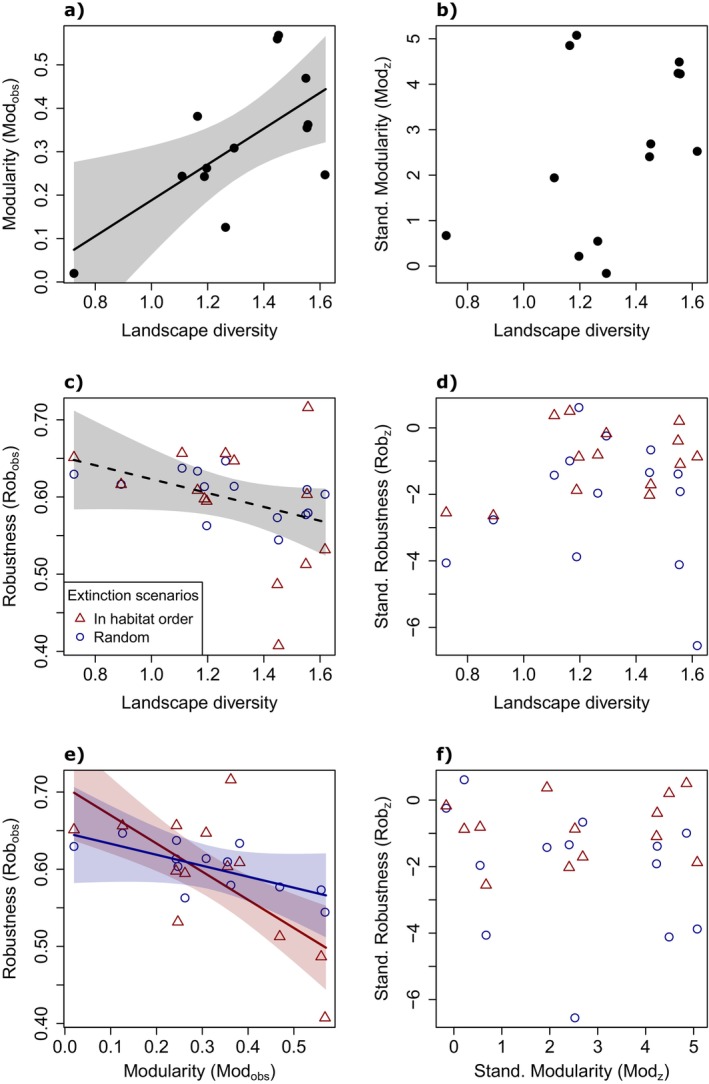
Overview of the observed and standardized network indices modularity and robustness, their relation to landscape diversity, and to each other. (a) Observed modularity and (b) standardized modularity relating to landscape diversity. (c) Observed robustness and (d) standardized robustness relating to landscape diversity. (e) Observed robustness relating to observed modularity. (f) Standardized robustness relating to standardized modularity. Raw data show robustness values for randomized extinctions (circle, blue) and extinctions in habitat order (triangle, red). Standardized z‐scores are calculated based on 1000 null networks for each landscape (z = (obs – null mean)/(null sd)). The steeper decreasing line in panel e refers to the extinction scenario ‘in habitat order’.

To do so, we calculated the observed modularity (Mod_obs_) of the landscape‐level bee‐habitat networks using function networklevel with index modularity from R package bipartite (Dormann & Strauss, 2014). To exclude possible confounding effects of different network sizes, we standardized the modularity values with z‐scores by generating 1000 random null networks for each landscape (function nullmodel() using Patefield's Algorithm; Dormann et al., 2008). We then calculated the network indices for all null networks and extracted their means (null mean) and standard deviations (null sd). Z‐scores were then calculated as (obs–null mean)/(null sd). As the network for landscape L5 only contained one habitat type, it was not possible to compute modularity z‐scores for this landscape. Consequently, L5 was not included in any statistics involving modularity. We then assessed the influence of landscape diversity on the networks' observed modularity (Mod_obs_) and their z‐scores (Mod_z_) using two linear models (Figure 5).

Using abundance data from pan trap sampling has been criticized (Tronstad et al., 2022). To check for the reliability of our abundance data for our weighted networks, we also calculated z‐scores for binary networks (i.e., using only presence–absence data) and also applied the same linear model. We did not find a different result for binary networks (see Results section), indicating that abundances in weighted networks did not skew the results.

#### Robustness of bee‐habitat networks to habitat loss

**FIGURE 6:**
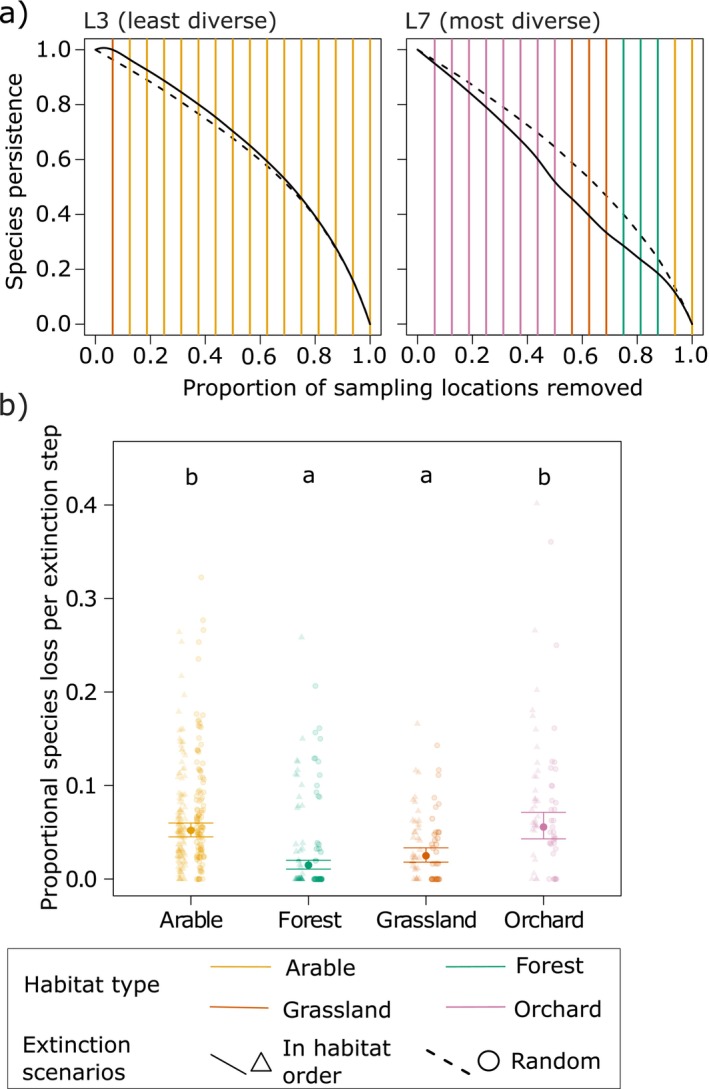
Robustness analysis of habitat types and landscapes. (a) The two example landscapes represent the landscapes with the lowest and highest landscape diversity. The solid extinction curves indicate the proportion of remaining species at each extinction step when the habitats die out in the order of orchard, grassland, forest, arable. The dashed lines represent completely randomized extinctions (both extinction curves are based on means per extinction step after 1000 repetitions). Robustness curves for all 14 landscapes can be found in Appendix S1: Figure S6. (b) The habitat type associated with an extinct sampling location influences the proportional species loss per extinction step (= species persistence at extinction step *x* minus species persistence at extinction step *x* + 1). Raw data are displayed as scattered, transparent points for extinctions in habitat order (triangle, left stack of points) and randomized extinctions (circle, right). Boxplots indicate model predictions and their 95% CIs. Letters indicate significant differences between habitat types.

We used a simulation approach based on primary habitat and secondary bee species extinctions to analyze the consequences of habitat loss for bee species (Dunne et al., 2002; Evans et al., 2013; Memmott et al., 2004). For the simulation, a custom extinction function was implemented to sequentially remove sampling locations from the network and monitor the effects on the remaining species. The input networks were based on landscape‐level matrices with sampling locations as rows, bee species as columns and their corresponding abundance as matrix fills. We wanted to simulate two extinction scenarios by initially shuffling these matrices in two different ways: (i) completely randomized, (ii) keeping the locations in a specific order of habitat types (1) orchard, (2) grassland, (3) forest, (4) arable land; method: (Evans et al., 2013), while locations within each habitat type were randomly shuffled with each repetition (e.g., if multiple orchard sampling locations were present, the order of their extinctions was randomized; after all orchard locations were extinct, the grassland locations followed in a similar way). We tested this specific habitat order as it is the socioeconomically most relevant scenario for our study regions, where traditional orchards vanish quickly and can be replaced by intensively managed grassland or arable land. During the extinction process, a single sampling location was removed from the network at each extinction step in a deterministic order, always starting with the first row. In total, 16 extinction steps were performed due to the 16 sampling locations per landscape. For each extinction step, we calculated the proportion of species lost and always recorded the sampling location and its habitat identity (Figure 6a; Appendix S1: Figure S6). We repeated the extinction process for both extinction scenarios (randomized, in habitat order) for 1000 times for each of the study landscapes.

In the first analysis, we assessed how habitat loss by habitat type or by extinction scenario (randomized, in habitat order) translated into the proportional bee species loss. To do so, we averaged the proportional species loss per sampling location over all repetitions of each extinction scenario. In the statistical model, we included this mean proportional species loss as a function of the habitat type, the extinction scenario and landscape diversity. To fit model assumptions, we needed to log‐transform the response variable and added a constant of 0.01 due to zeros in the dataset. Using ANOVA with F‐tests, we performed stepwise backward model selection to identify the most parsimonious model and the statistical significance of explanatory variables. For the best model, we calculated predictions and their 95% CIs of the proportional species loss per extinction step and habitat type.

For a second analysis, we assessed the robustness of the species‐habitat network to the simulated loss of habitats for both extinction scenarios (randomized, in habitat order). To achieve that, we averaged the proportional species loss for each extinction step over all repetitions, separately for each extinction scenario. Using these values, we fitted extinction curves (R function splinefun) and calculated their area under the curve (R function integrate; standardized between 0 and 1, with 0 indicating low and 1 high robustness) for each landscape and extinction scenario (Figure 6a; Appendix S1: Figure S6). These values then referred to the observed robustness of the networks (Rob_obs_). Just as for modularity, we also calculated z‐scores (Rob_z_) for robustness based on 1000 random null networks for all 14 landscapes and both extinction scenarios to which we applied the same protocol as for calculating the observed robustness. To understand if the application of a certain order of habitat extinctions and the landscape diversity played a role for Rob_obs_ and Rob_z_, we ran a linear mixed‐effects model for both response variables (R package lme4). These models assessed the influence of the two extinction scenarios (random vs. in habitat order) and the landscape diversity, as well as their interaction, on the response variables and included a random effect of landscape ID. Using ANOVA with Chi‐squared tests, we performed stepwise backward model selection to identify the most parsimonious model and the statistical significance of explanatory variables.

Lastly, we wanted to know how network robustness (averaged per landscape over both extinction scenarios) and network modularity related to each other. To test this, we assessed similar linear mixed‐effects models for observed and standardized robustness as above but replacing landscape diversity as an explanatory variable by observed or standardized modularity. A similar model was also analyzed for the observed modularity from binary networks. All figures and statistical analyses were done in R version 4.4.1 (R Core Team, 2024) with packages bipartite (Dormann et al., 2008), effects (Fox & Weisberg, 2018), emmeans (Lenth, 2024), ggeffects (Lüdecke, 2018), ggplot2 (Wickham, 2016), glmmTMB (Brooks et al., 2017), iNEXT (Chao et al., 2014; Hsieh et al., 2024), lme4 (Bates et al., 2015), multcomp (Hothorn et al., 2008), and vegan (Oksanen et al., 2024).

## RESULTS

We collected 950 wild bee individuals (i.e., excluding *Apis mellifera*) from 75 species across the 14 study landscapes. Of the 224 sampling locations across these landscapes, 110 were mainly associated with arable land, 44 with forest, 33 with grassland, 34 with orchard, and 3 with settlements (Figure 2a). The most diverse bee genera were *Andrena* (25 species), *Lasioglossum* (16), *Bombus* (10), and *Halictus* (6). The most abundant bee species were *Andrena flavipes* (135 individuals), *Lasioglossum zonulum* (128), and *Lasioglossum malachurum* (114). In arable land, we collected 535 individuals across 59 species, in grassland 64 individuals across 27 species, in forest 125 individuals across 31 species and in orchard 209 individuals across 41 species (Figure 2b). Bee species richness in the study landscapes ranged from 16 to 31 species (mean ± sd = 22.4 ± 4.3) and did not correlate with landscape diversity (Pearson's *r* = 0.23, *p* = 0.424). Sampling completeness of bee species richness varied between landscapes (mean ± sd = 56% ± 15%) but did not correlate with landscape diversity (Pearson's *r* = −0.30, *p* = 0.292). Similarly, the completeness of sampled interactions in the networks was 50% ± 13% (mean ± sd) but decreased with increasing landscape diversity (Pearson's *r* = −0.647, *p* = 0.0125).

### Habitat preferences of wild bees

Wild bees showed a preference for orchards, as indicated by their higher densities than expected from the proportional cover of orchards within landscapes (mean = 0.122; 95% CI: 0.019–0.225; Figure 3b). Similarly, higher bee densities were found than expected from arable land cover (0.100; 0.002–0.199). In contrast, the proportion of wild bees in grasslands corresponded to the cover of the habitat in the landscape (−0.033; −0.075 to 0.010; Figure 3b). Forests showed a lower bee density despite a larger proportional habitat cover (−0.147; −0.269 to −0.024), indicating that bees avoided forests. These patterns were confirmed by the probabilistic networks, apart from the higher‐than‐expected densities on arable land (Appendix S1: Figure S5).

### Wild bee richness and functional composition

For wild bee species richness per sampling location, only the habitat type played a relevant role ($\chi_{3}^{2}$ = 45.12, *p* < 0.001; Figure 3c). Irrelevant factors were habitat proportion (*p* = 0.678) and landscape diversity (*p* = 0.543) as well as the latter's interaction with habitat type (*p* = 0.551). Species richness was highest in orchard (mean = 3.7 species; 95% CI: 2.8–4.7 species), followed by grassland (2.2; 1.6–3.0) and forest (1.1; 0.8–1.5). Arable land showed a similarly high species richness to orchards and grassland (3.2; 2.7–3.8).

The functional composition of bee assemblages differed among habitat types when considering sociality ($\chi_{3}^{2}$ = 8.31, *p* = 0.040) and dietary breadth of wild bees ($\chi_{3}^{2}$ = 10.46, *p* = 0.015; Figure 4). Thus, the probability of wild bees being social was higher in forest (57%) than in grassland (33%), while orchard and arable land showed an intermediate probability (37% and 36%). The share of specialized oligolectic bees was significantly higher in orchards (i.e., 11%; 89% polylectic) than in arable land (4% oligolectic; 96% polylectic). Dietary breadth of bees in forest and grassland ranged between these two. Habitat type did not significantly influence the average body size ($\chi_{3}^{2}$ = 3.28, *p* = 0.351) or the probability of bees being endangered ($\chi_{3}^{2}$ = 2.19, *p* = 0.534). Overall, neither the interaction between habitat type and landscape diversity, nor the additive effects of habitat proportion or landscape diversity were significant in any of the models applied to the four different traits.

### Modularity of bee‐habitat networks

The modularity of the bee‐habitat networks (Mod_obs_) increased with increasing landscape diversity (estimate = 0.413, *p* = 0.015; Figure 5a). However, when modularity was standardized for network size (Mod_z_), the influence of landscape diversity disappeared (estimate = 3.027, *p* = 0.171; Figure 5b). Network size ranged between 24 and 155 (mean ± sd = 73.6 ± 32.4) and correlated with landscape diversity (Pearson's *r* = 0.751, *p* = 0.002; Appendix S1: Figure S7). Similarly, standardized modularity for binary species‐habitat networks was also not influenced by landscape diversity (estimate = −0.622, *p* = 0.434), indicating that our abundance data from pan traps did not skew results.

### Robustness of bee‐habitat networks to habitat loss

Our simulation of habitat loss in the bee‐habitat networks revealed differences between habitat types in the proportion of bee species lost per extinction step (*F*_3,438_ = 27.05, *p* < 0.001; Figure 6b). However, neither the extinction scenario (randomized or in habitat order; *p* = 0.493) nor the landscape diversity (*p* = 0.074) mattered for the proportion of species lost. On average, the simulated loss of sampling locations within arable land (estimate = 5.2%; 95% CI: 4.5%–6.0%) or orchard (5.6%; 4.3%–7.1%) resulted in a ca. 5% reduction in species richness in the study landscape. By contrast, removing one forest (1.5%; 1.1%–2.0%) or grassland location (2.5%; 1.8%–3.3%) led to a significantly lower species loss of ca. 2% on average. In extreme cases, the loss of one location could lead to a much higher species loss in arable land (32.3%) or orchard (40.2%) while the loss of grassland and forest locations was not as variable and remained even in extreme situations below 26% species loss.

The observed robustness of bee‐habitat networks to simulated habitat extinctions (Rob_obs_) ranged between 0.41 and 0.72. Rob_obs_ was marginally significantly negatively influenced by landscape diversity (estimate = −0.090, *p* = 0.074; Figure 5c), but did not differ between extinction scenarios, that is, if habitats were removed randomly or in a specific order (*p* = 0.519). Standardized network robustness using z‐scores (Rob_z_) was not related to landscape diversity (estimate = 0.560, *p* = 0.661; Figure 5d). However, applying a random extinction scenario led to lower z‐scores by 1.2 than habitat extinctions in their specific order (*p* = 0.022).

Assessing the influence of modularity on robustness, observed values had a negative impact (modularity estimate = −0.365, *p* < 0.001). There was an interaction with the extinction scenario (*p* = 0.049), showing that this negative effect was less pronounced for the random extinction scenario (Figure 5e). Considering z‐scores of both metrics, there was no influence of modularity on robustness (estimate: −0.106, *p* = 0.585), but Rob_z_ values for random habitat extinctions were generally lower than in the case of extinctions in habitat order (*p* = 0.021; Figure 5f). A sensitivity analysis using binary networks showed similar results, but without any interaction effects (for observed values: Mod_obs_ estimate = −0.302, *p* = 0.004; for z‐scores: Mod_z_ estimate = 0.093, *p* = 0.865).

## DISCUSSION

Here we studied how habitat complementarity and landscape diversity affect wild bee diversity and the robustness of bee‐habitat networks in agricultural landscapes. Our landscape‐wide sampling revealed that wild bees favored orchards, followed by arable land and grasslands, while they avoided forests. In particular, orchards were important for supporting high bee species richness and solitary oligolectic (i.e., specialized) bee species, while social bees were more likely to be found in forests. While the observed modularity of the networks increased with increasing landscape diversity, correcting modularity for network size (using z‐scores) let this effect disappear. Observed network robustness tended to decrease with landscape diversity, while robustness z‐scores did not. Network robustness did not differ between extinction scenarios in which the different habitats were removed randomly or according to their sensitivity to agricultural intensification.

### Relative importance of habitat types for bees, their richness and functional composition

Identifying landscape designs for bee conservation requires assessments that cover all major habitat types of agricultural landscapes (Scherber et al., 2019). Applying the landscape‐level sampling approach proposed by Scherber et al. (2019), we identified key habitats for wild bee conservation. Wild bees were disproportionately represented in orchards with significantly higher numbers than expected from each study landscape's orchard areas. Orchards also harbored the highest bee species richness. These results suggest the importance of extensively managed, traditional land use systems for bee conservation. The traditional orchards in our study were characterized by their old trees and extensive grassland management, which can result in a high number of nesting opportunities above and below ground, and a diverse, richly flowering undergrowth (Guariento et al., 2024). In fact, among the studied habitat types, orchards had the highest density and diversity of flowering plants (11× more species and 14× more floral units than forests; Appendix S1: Section S1). As a result, orchards seemed to provide critical resources for oligolectic bee species such as *Osmia florisomnis* (specialized on Ranunculaceae), *Eucera nigrescens* (specialized on Fabaceae, especially *Vicia sepium*), and *Andrena lathyri* (specialized on *Vicia* sp. and *Lathyrus* sp.; Westrich, 2019). The traditional orchards in Southern Germany have strongly decreased in area and quality in the past >20 years (Breunig et al., 2020), despite being a UNESCO Intangible Cultural Heritage (German Commission for UNESCO, 2023) and being protected under the German Nature Conservation Act since 2022 (§30). High opportunity costs and economic incentives result in abandonment and conversion towards more profitable land uses (Plieninger et al., 2015). Policies that subsidize bee‐friendly cultivation or reward certified premium products could represent an economically attractive alternative to preserving and maintaining this ecologically important bee habitat (Guariento et al., 2024).

Similar to orchards, arable land exhibited higher wild bee densities than expected; however, this pattern was not reflected in the probabilistic assignment of bees to their habitat. This suggests that bees at least do not actively avoid arable land. Given that arable land is predominantly characterized by cereal crops, which provide limited forage resources for wild bees, the observed densities may be driven by the presence of flowering crops (e.g., fava beans, soy) within the study landscapes. However, typical mass‐flowering crops such as oilseed rape (Riedinger et al., 2015) played only a minor role in terms of area in our study region (mean ± sd = 2.7% ± 3.9%). In addition, small seminatural structures, such as hedges, located between arable fields can promote the benefits of crop heterogeneity and thus support diverse wild bee communities in arable areas (Tassoni et al., 2024). Grasslands, by contrast, did not attract more bees than expected from their cover and supported less species than orchards. This finding may appear surprising, given that many studies emphasize the high value of extensively managed grasslands for bee conservation (Jauker et al., 2013; Klaus et al., 2021). However, most grassland areas in our study region are meadows for silage production, dominated by few grass species and managed with high rates of fertilizer application and frequent mowing (Hartmann, 2012). Consequently, these grasslands offer little food or nesting opportunities for specialized bees (Ekroos et al., 2020).

Forests had the lowest bee densities and richness of all habitats. Most bee species may have avoided closed forest areas (Roberts et al., 2017; Westrich, 2019), which were also low in flowering resources (only two flowering units per square meter; Appendix S1: Section S1). Furthermore, old trees and deadwood that could serve as nesting sites were rarely present, indicating rather intensive management (personal observation). However, forests harbored the highest density of social wild bees. These included, for example, *Bombus pratorum* and *Lasioglossum calceatum*, which are social forest bees (Westrich, 2019). Due to their communication structures, social bees may also be at an advantage to solitary bees in exploiting the few floral resources in forests compared to open habitats (Rutschmann et al., 2023). Furthermore, bumble bees, as the most important social bees in our study region, show their highest activity at much lower temperatures than most solitary bees (Fründ et al., 2013). Thus, the cooler microclimate in forests might be avoided by solitary bees.

In general, our findings regarding the relative importance of habitats, for example, for nesting or based on their potential provision of food sources for wild bees, should be interpreted with caution. This is particularly true given that, with an estimated sample completeness of approximately 50%, we have most likely missed many important species. These are likely to include rare species, which are often habitat and/or resource specialists (Westrich, 2019). It is therefore possible that even more habitat specialists would have been discovered with greater sampling effort. At the same time, the habitat preference of some species may have been overestimated if they could only be found in one or a few habitats but in fact had colonized more.

Despite differences in habitat use and species richness, we found no difference in the proportion of endangered and non‐endangered bee species between habitats. It is possible that most of the highly endangered bee species had already been filtered out of our study landscapes. The 75 wild bee species in our study can be characterized as predominantly generalist species. Nevertheless, generalists can also be threatened (Schwenninger et al., 2025), which means that agricultural landscapes must also become the focus of bee conservation efforts. Our study shows that all habitats should be included when it comes to protecting a high diversity of bee species.

### Species‐habitat networks inform habitat complementarity and landscape diversity for bees

Most conservation measures treat habitats and their associated species as separate entities. However, in diverse landscapes, the habitats and their species communities are connected in metacommunities, for example, when species use several habitats simultaneously (Marini et al., 2019). Analyzing bee‐habitat networks makes it possible to understand the structure of these landscape‐wide associations and to derive consequences for landscape management.

Simulating the loss of habitats, we found that the loss of traditional orchards or arable land led to a higher proportional species loss than grassland or forest loss. This seems to be a logical consequence of the relative importance of the habitat types for the wild bees and bee species. However, it was interesting that the extinction scenario—that is, whether habitat loss occurred randomly or following a likely intensification scenario, with orchards being lost first, and arable land last—did not affect the proportional species loss. Consequently, it makes no difference at what point in time a specific habitat is lost. Orchards, for example, should therefore always be protected, even if other habitats remain.

The observed modularity of bee‐habitat networks increased with greater landscape diversity, indicating that with a greater number of habitat types, these complemented each other in supporting bees in diverse landscapes. However, when correcting modularity for network size (z‐scores), the previously observed correlation with landscape diversity was no longer significant. Similarly, the marginal significant decrease in robustness with higher landscape diversity disappeared when applying z‐scores to robustness. This indicates that the increase in modularity and the marginal decrease in robustness appeared to be primarily driven by increases in network size. This assumption is supported by the fact that network size (i.e., the number of possible species‐habitat links) increased with increasing landscape diversity. Since we undersampled interactions in diverse landscapes more than in simple landscapes, a larger sample size would likely have resulted in even higher observed modularity in diverse landscapes compared to simpler landscapes. Therefore, we do not expect these results to change, even if we had taken more samples. In general, the sampling completeness of interactions of circa 50% matches values from other network studies (e.g., Chacoff et al., 2012; Grass et al., 2018). Nevertheless, further studies with an even greater sampling effort are needed to examine the transferability of these results to other contexts.

In our study, observed modularity and network robustness were negatively related to each other. Consequently, more modular bee‐habitat networks in more diverse landscapes tended to be less robust than their counterparts in arable dominated landscapes with less modular networks. For standardized modularity and robustness, this relationship disappeared. This is contrary to others who found that the modularity of networks positively influences their robustness (Gilarranz et al., 2017). There, higher modularity could buffer networks against habitat loss if habitats form discrete modules, thereby limiting extinction events to the affected modules and not propagating them through the rest of the network (Martins et al., 2024). A possible explanation for our findings is that species‐habitat networks with low modularity could result from landscape simplification where only a few habitats (e.g., arable land) and an impoverished species pool (e.g., of generalists) remain (i.e., low network sizes in landscapes of low habitat diversity). The remaining network core may prove more robust than a more complex network composed of a variety of habitats and species (see also studies on plant–pollinator networks and habitat fragmentation: e.g., Grass et al., 2018). Hence, greater landscape diversity and modularity do not necessarily buffer against the effects of habitat loss.

## CONCLUSIONS

Our study shows that analyzing bee‐habitat networks provides crucial insights into the relative importance of habitats for bee conservation in diverse agricultural landscapes. We show that different habitats harbor different parts of bee diversity and that even in diverse agricultural landscapes, bee diversity can be highly vulnerable to habitat loss. Conservation efforts and suitable policy measures must therefore explicitly consider the landscape scale. Such measures need to recognize that the protection of biodiversity requires the maintenance and conservation of habitat diversity, including both traditional extensively managed land uses and intensively managed areas.

## AUTHOR CONTRIBUTIONS

Marit Kinga Kasten and Ingo Grass conceived the ideas and designed the methodology; Marit Kinga Kasten and Sara Tassoni collected the data; Marit Kinga Kasten, Ingo Grass, and Thomas Hiller analyzed the data; Marit Kinga Kasten and Ingo Grass led the writing of the manuscript. All authors contributed critically to the drafts and gave final approval for publication.

## CONFLICT OF INTEREST STATEMENT

The authors declare no conflicts of interest.

## Supporting information


Appendix S1.

## Data Availability

Data (Kasten et al., 2026) are available in Dryad at https://doi.org/10.5061/dryad.s7h44j1p7.
