# Supplementary material for: Species‐habitat networks reveal key habitats for landscape‐level wild bee conservation
Source: Ecol Appl. 2026 Apr 1;36(3):e70224. doi: 10.1002/eap.70224 (PMC13044500; doi:10.1002/eap.70224)
Supplement: Supplementary file 1 — Appendix S1. [file EAP-36-e70224-s001.pdf]

Appendix S1

Species-habitat networks reveal key habitats for landscape-level wild bee conservation

Marit Kinga Kasten, Sara Tassoni, Thomas Hiller, Markus Röhl, Michael Roth, Ingo Grass

Ecological Applications

Contents

Figure S1: Impression of habitat types

Figure S2: Habitat composition of all 14 landscapes

Figure S3: Total area and number of sampling locations for each habitat type

Figure S4: Landscape-level species-habitat networks of all 14 landscapes

Figure S5: Habitat preference and avoidance based on probabilistic species-habitat networks

Figure S6: Robustness analysis of all 14 landscapes

Figure S7: Size of the species-habitat networks for landscapes of differing diversity

Section S1: Floral composition

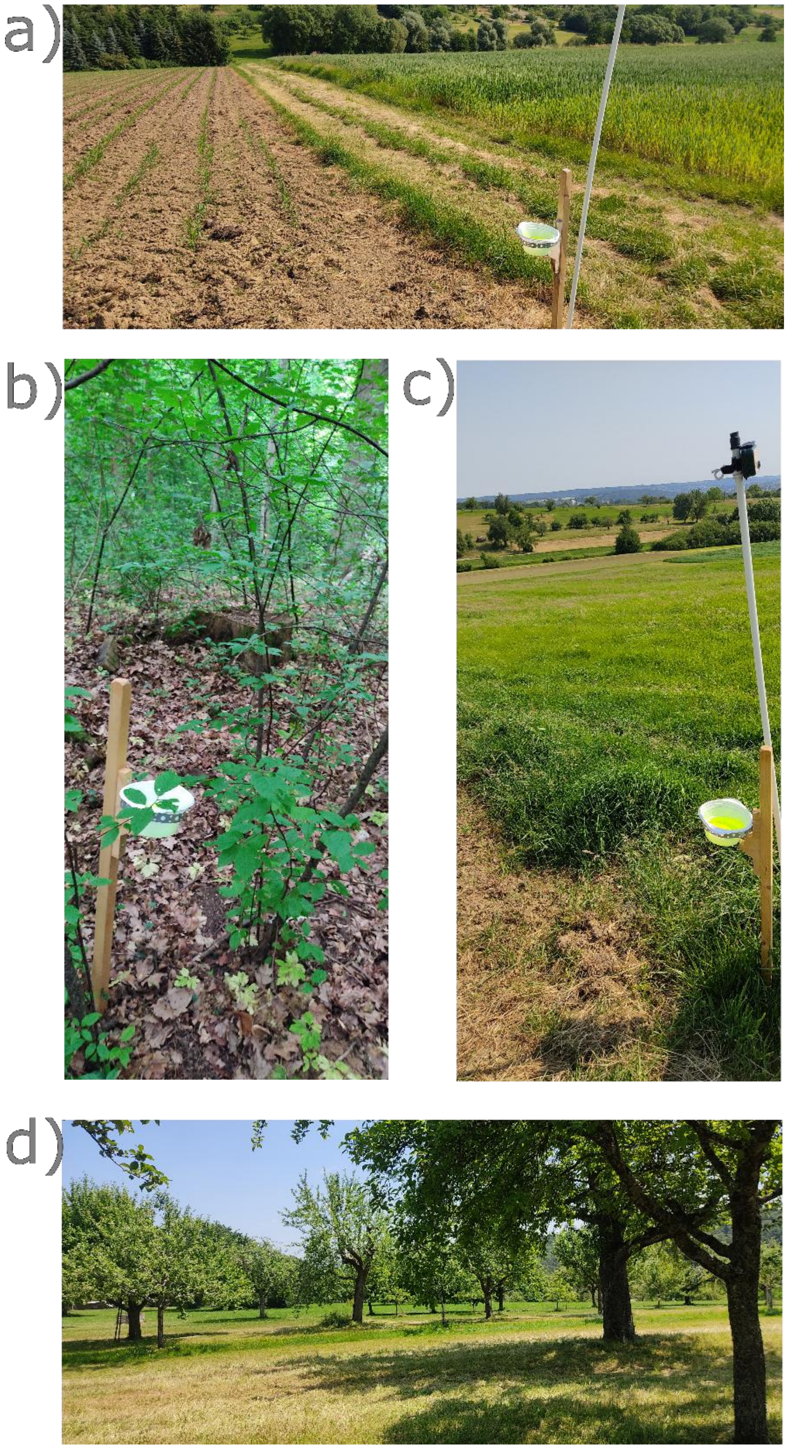

Figure S1: Impressions of the four habitat types in the study landscapes: a) arable, b) forest, c) grassland, d) orchard. Photos by Carlos Gonzalez

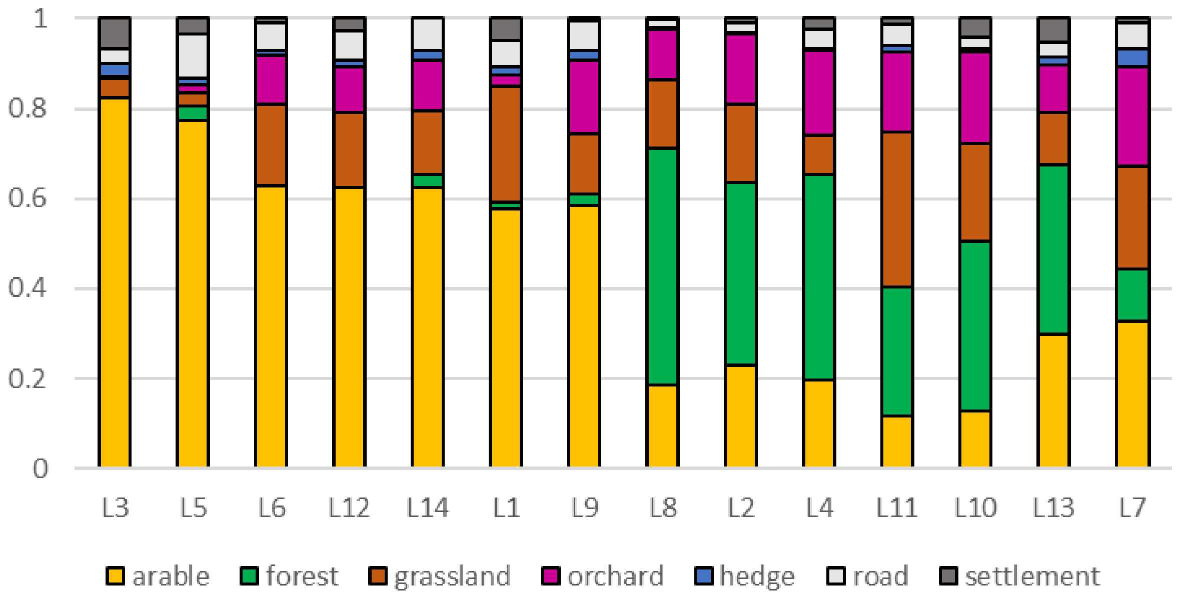

Figure S2: Habitat composition of all 14 landscapes at the landscape scale, sorted by increasing landscape diversity. Displayed are the four major habitat types arable land (yellow), forest (green), grassland (brown), orchard (purple) and minor habitat types (hedge: dark blue, road: light grey, settlement: dark grey).

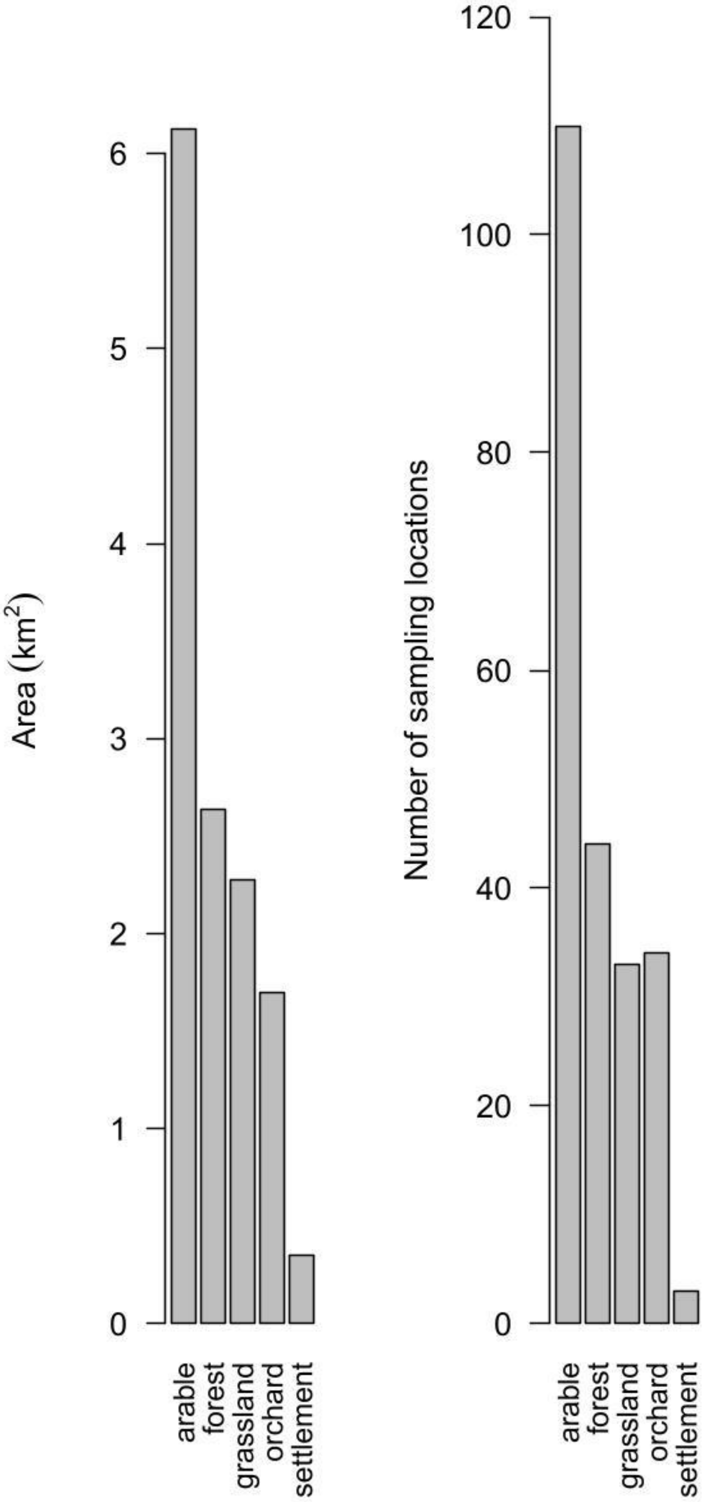

Figure S3: (a) Total area and (b) number of sampling locations for each habitat type, both summed across all 14 landscapes. The portion of a habitat type's total area present over all landscapes corresponded with their portion of sampling locations. For instance, arable land was present on 6.13 km^2 among all 14 landscapes and was assigned 110 times as habitat type (figure similar to Scherber et al., 2019).

Figure S4: Landscape-level species-habitat networks of all 14 landscapes.

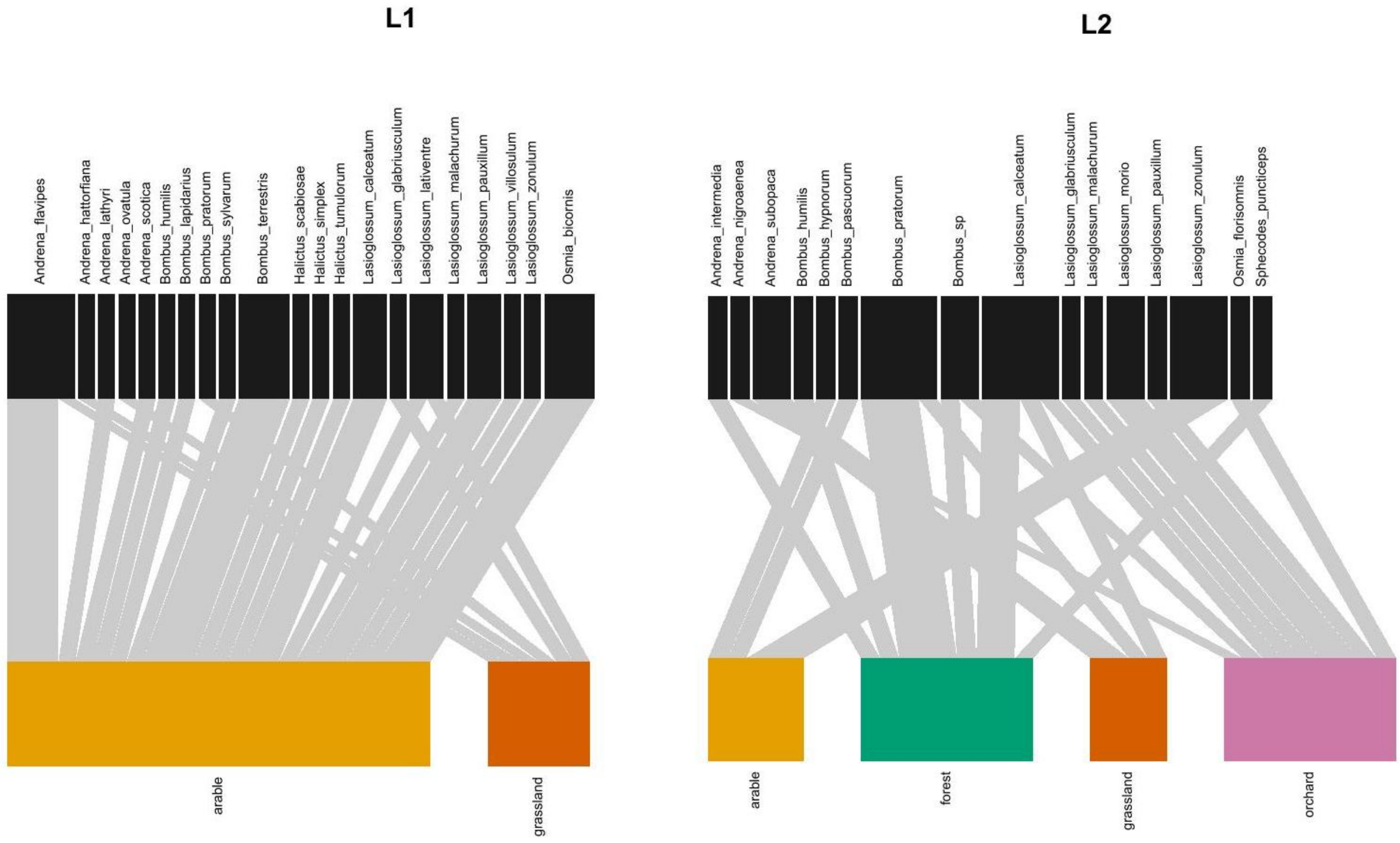

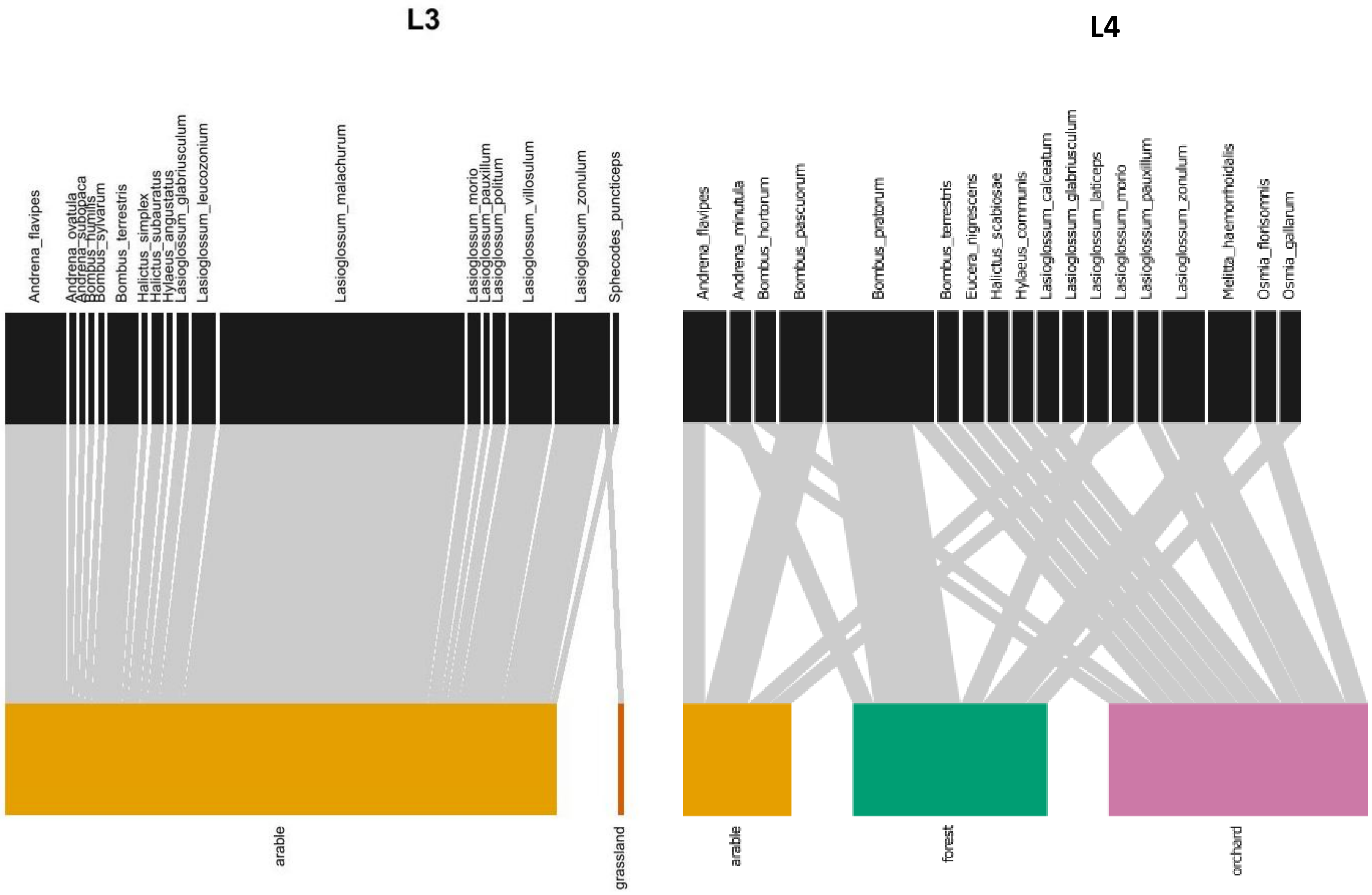

L3
Andrena_flavipes
Andrena_ovatula
Andrena_subopaca
Bombus_humilis
Bombus_sylvarum
Bombus_terrestris
Halictus_simplex
Halictus_subauratus
Hylaeus_angustatus
Lasioglossum_glabriusculum
Lasioglossum_leucozonium
Lasioglossum_malachurum
Lasioglossum_morio
Lasioglossum_pauxillum
Lasioglossum_politum
Lasioglossum_villosulum
Lasioglossum_zonulum
Sphecodes_puncticeps
arable
grassland
L4
Andrena_flavipes
Andrena_minutula
Bombus_hortorum
Bombus_pascuorum
Bombus_pratorum
Bombus_terrestris
Eucera_nigrescens
Halictus_scabiosae
Hylaeus_communis
Lasioglossum_calceatum
Lasioglossum_glabriusculum
Lasioglossum_laticeps
Lasioglossum_morio
Lasioglossum_pauxillum
Lasioglossum_zonulum
Melitta_haemorrhoidalis
Osmia_florisomnis
Osmia_gallarum
arable
forest
orchard

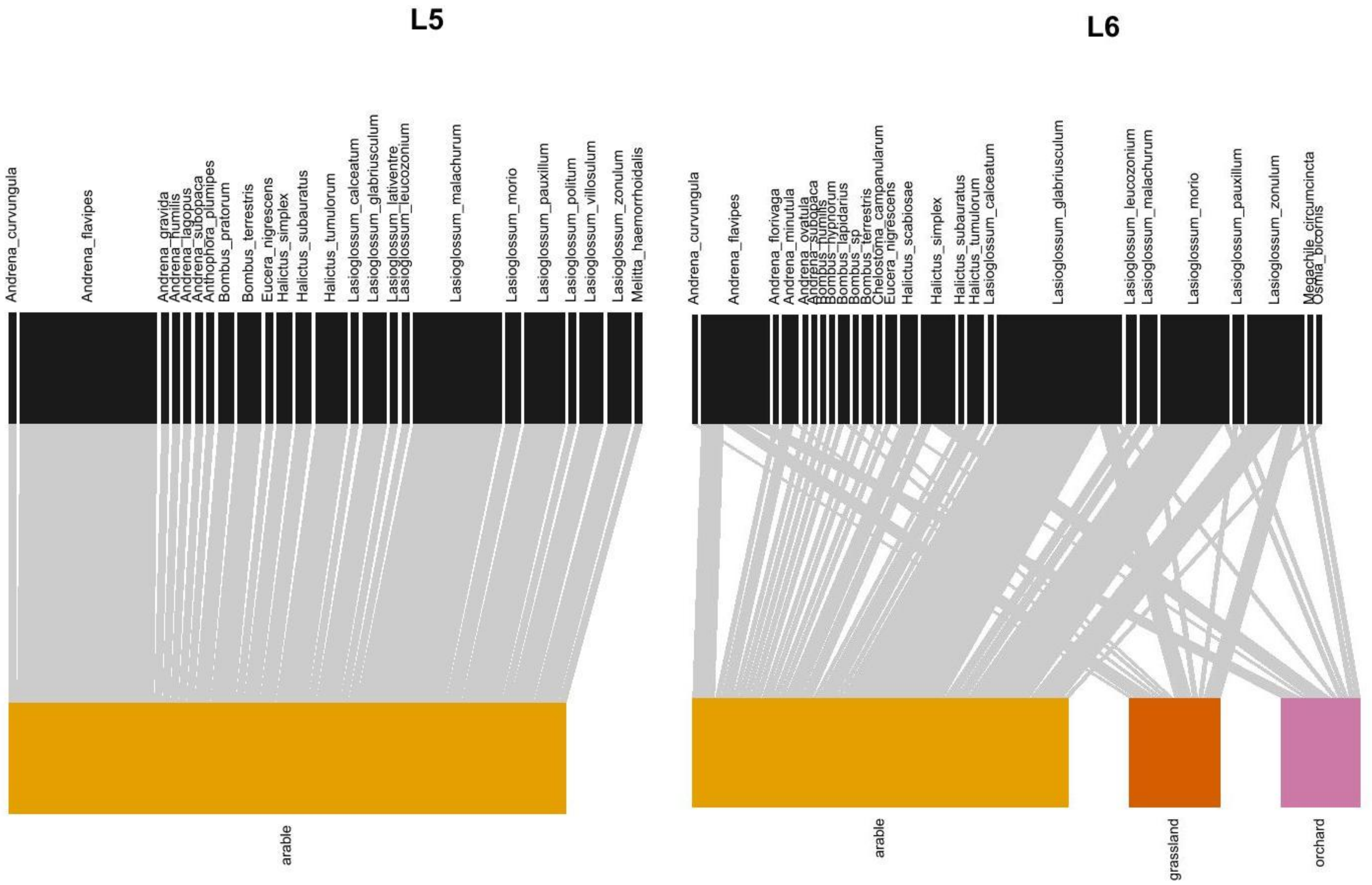
L5
Andrena_curvungula
Andrena_flavipes
Andrena_gravida
Andrena_humilis
Andrena_lagopus
Andrena_subopaca
Anthophora_plumipes
Bombus_pratorum
Bombus_terrestris
Eucera_nigrescens
Halictus_simplex
Halictus_subauratus
Halictus_tumulorum
Lasioglossum_calceatum
Lasioglossum_glabriusculum
Lasioglossum_lativentre
Lasioglossum_leucozonium
Lasioglossum_malachurum
Lasioglossum_morio
Lasioglossum_pauxillum
Lasioglossum_politum
Lasioglossum_villosulum
Lasioglossum_zonulum
Melitta_haemorrhoidalis
arable
L6
Andrena_curvungula
Andrena_flavipes
Andrena_florivaga
Andrena_minutula
Andrena_ovatula
Andrena_subopaca
Bombus_humilis
Bombus_hypnorum
Bombus_lapidarius
Bombus_sp
Bombus_terrestris
Chelostoma_campanularum
Eucera_nigrescens
Halictus_scabiosae
Halictus_simplex
Halictus_subauratus
Halictus_tumulorum
Lasioglossum_calceatum
Lasioglossum_glabriusculum
Lasioglossum_leucozonium
Lasioglossum_malachurum
Lasioglossum_morio
Lasioglossum_pauxillum
Lasioglossum_zonulum
Megachile_circumcincta
Osmia_bicornis
arable
grassland
orchard

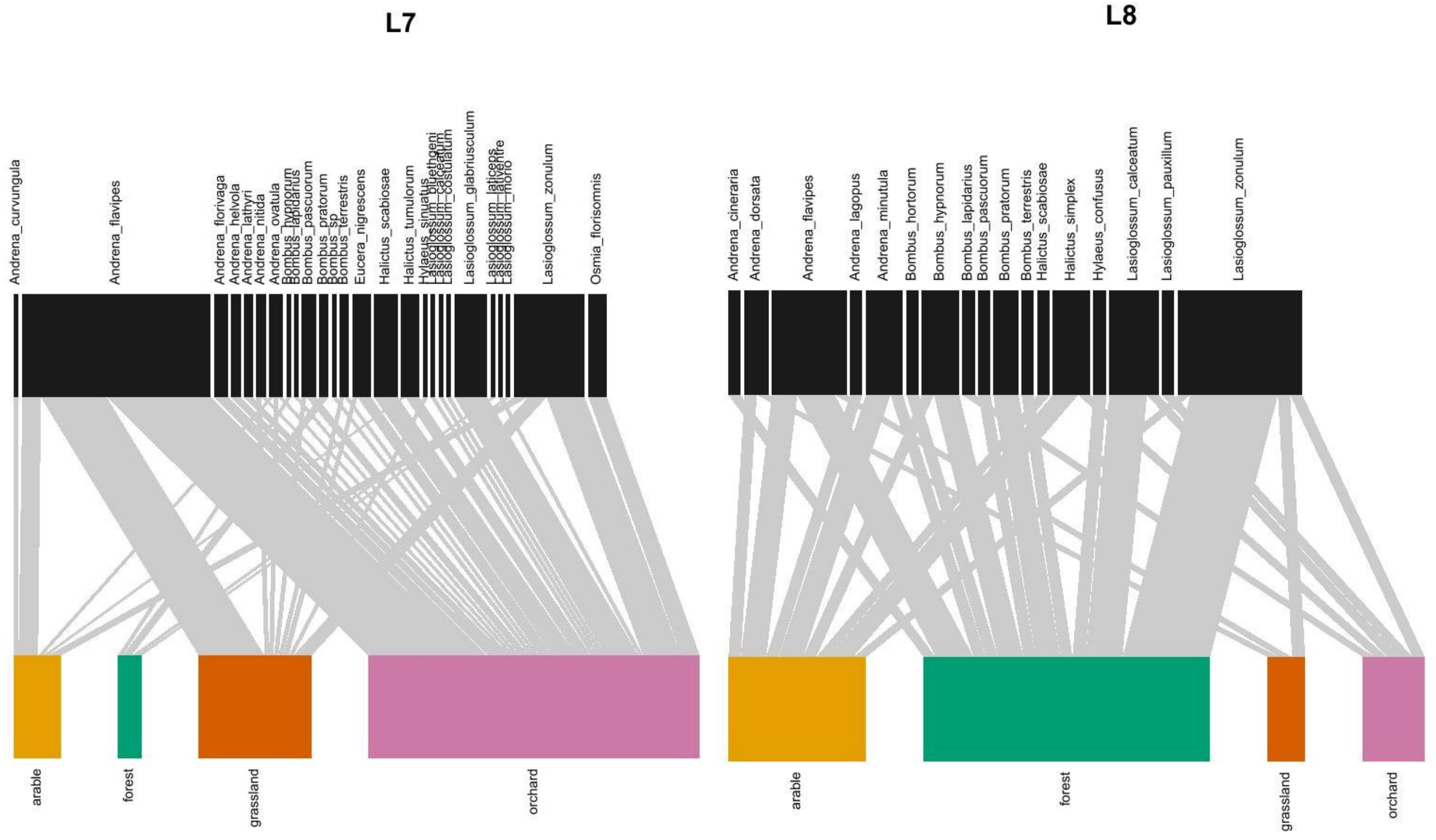
L7
Andrena_curvungula
Andrena_flavipes
Andrena_florivaga
Andrena_helvola
Andrena_lathyri
Andrena_nitida
Andrena_ovatula
Bombus_hypnorum
Bombus_lapidarius
Bombus_pascuorum
Bombus_pratorum
Bombus_sp
Bombus_terrestris
Eucera_nigrescens
Halictus_scabiosae
Halictus_tumulorum
Hylaeus_sinuatus
Lasioglossum_bluethgeni
Lasioglossum_calceatum
Lasioglossum_costulatum
Lasioglossum_glabriusculum
Lasioglossum_laticeps
Lasioglossum_lativentre
Lasioglossum_morio
Lasioglossum_zonulum
Osmia_florisomnis
arable
forest
grassland
orchard
L8
Andrena_cineraria
Andrena_dorsata
Andrena_flavipes
Andrena_lagopus
Andrena_minutula
Bombus_hortorum
Bombus_hypnorum
Bombus_lapidarius
Bombus_pascuorum
Bombus_pratorum
Bombus_terrestris
Halictus_scabiosae
Halictus_simplex
Hylaeus_confusus
Lasioglossum_calceatum
Lasioglossum_pauxillum
Lasioglossum_zonulum
arable
forest
grassland
orchard

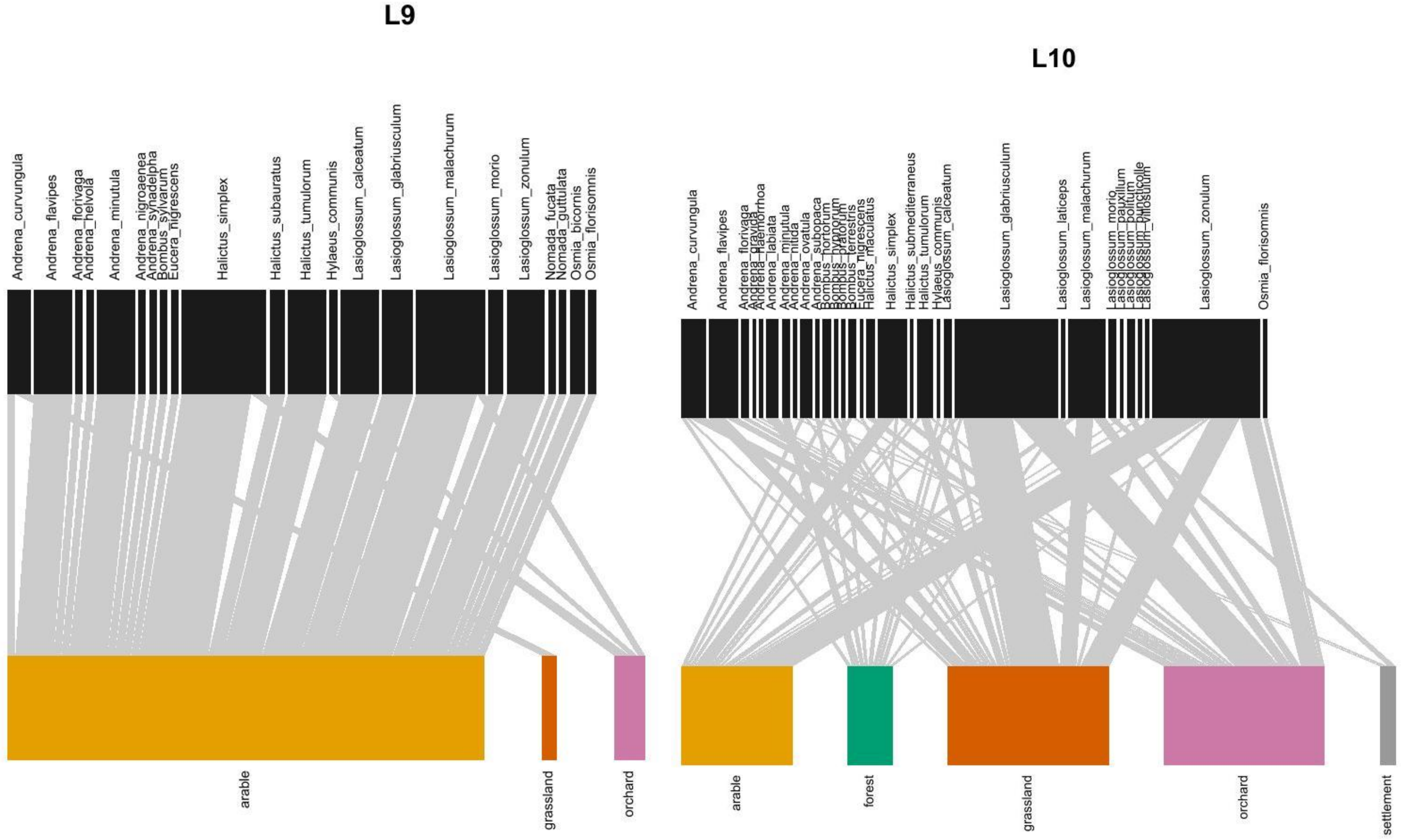
L9
Andrena_curvungula
Andrena_flavipes
Andrena_florivaga
Andrena_helvola
Andrena_minutula
Andrena_nigroaenea
Andrena_synadelpha
Bombus_sylvarum
Eucera_nigrescens
Halictus_simplex
Halictus_subauratus
Halictus_tumulorum
Hylaeus_communis
Lasioglossum_calceatum
Lasioglossum_glabriusculum
Lasioglossum_malachurum
Lasioglossum_morio
Lasioglossum_zonulum
Nomada_fucata
Nomada_guttulata
Osmia_bicornis
Osmia_florisomnis
arable
grassland
orchard
L10
Andrena_curvungula
Andrena_flavipes
Andrena_florivaga
Andrena_gravida
Andrena_haemorrhoa
Andrena_labiata
Andrena_minutula
Andrena_nitida
Andrena_ovatula
Andrena_subopaca
Bombus_hortorum
Bombus_terrestris
Eucera_nigrescens
Halictus_maculatus
Halictus_simplex
Halictus_submediterraneus
Halictus_tumulorum
Hylaeus_communis
Lasioglossum_calceatum
Lasioglossum_glabriusculum
Lasioglossum_laticeps
Lasioglossum_malachurum
Lasioglossum_morio
Lasioglossum_pauxillum
Lasioglossum_politum
Lasioglossum_villosulum
Lasioglossum_zonulum
Osmia_florisomnis
arable
forest
grassland
orchard
settlement

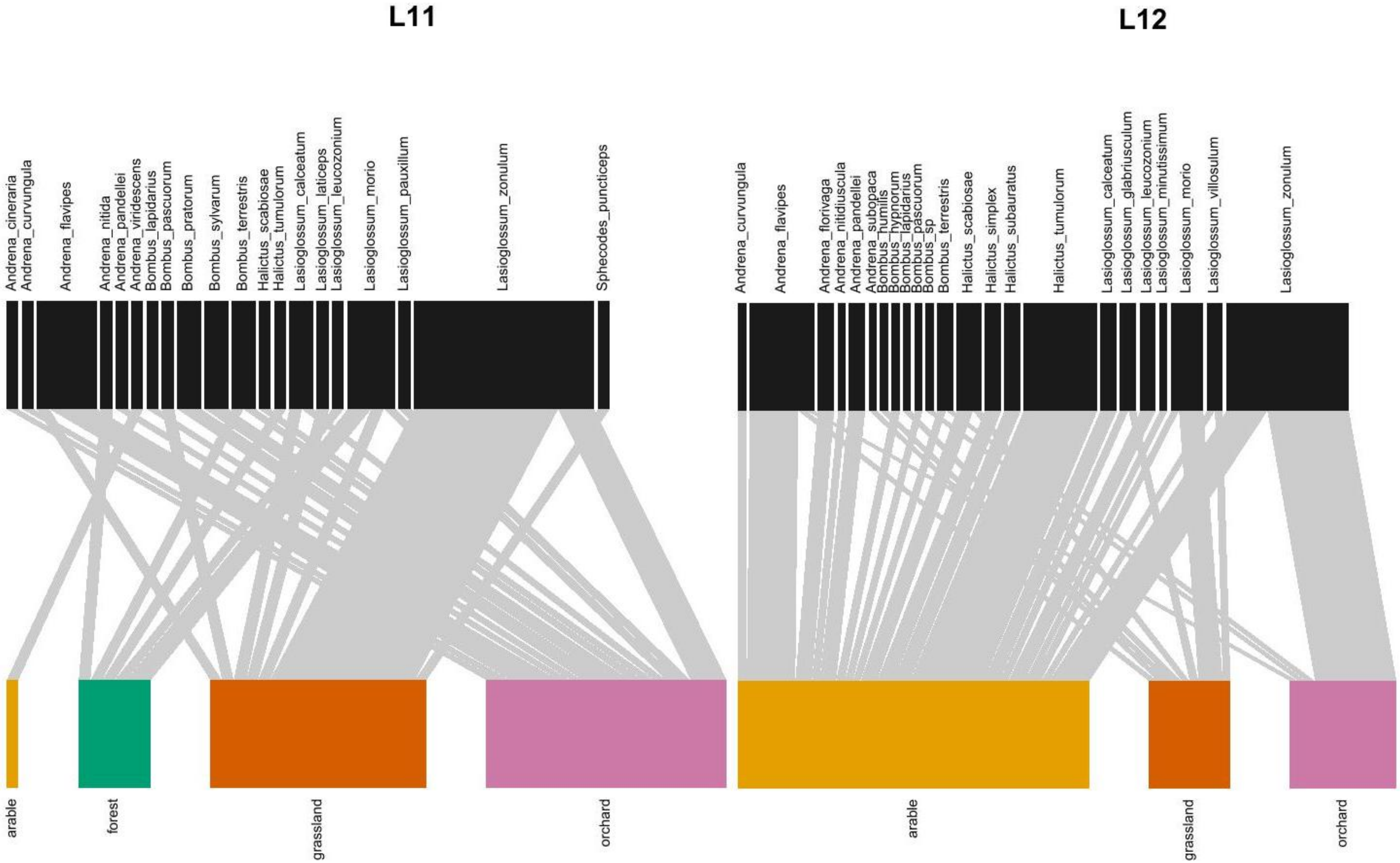
L11
Andrena_cineraria
Andrena_curvungula
Andrena_flavipes
Andrena_nitida
Andrena_pandellei
Andrena_viridescens
Bombus_lapidarius
Bombus_pascuorum
Bombus_pratorum
Bombus_sylvarum
Bombus_terrestris
Halictus_scabiosae
Halictus_tumulorum
Lasioglossum_calceatum
Lasioglossum_laticeps
Lasioglossum_leucozonium
Lasioglossum_morio
Lasioglossum_pauxillum
Lasioglossum_zonulum
Sphecodes_puncticeps
arable
forest
grassland
orchard
L12
Andrena_curvungula
Andrena_flavipes
Andrena_florivaga
Andrena_nitidiuscula
Andrena_pandellei
Andrena_subopaca
Bombus_humilis
Bombus_hypnorum
Bombus_lapidarius
Bombus_pascuorum
Bombus_sp
Bombus_terrestris
Halictus_scabiosae
Halictus_simplex
Halictus_subauratus
Halictus_tumulorum
Lasioglossum_calceatum
Lasioglossum_glabriusculum
Lasioglossum_leucozonium
Lasioglossum_minutissimum
Lasioglossum_morio
Lasioglossum_villosulum
Lasioglossum_zonulum
arable
grassland
orchard

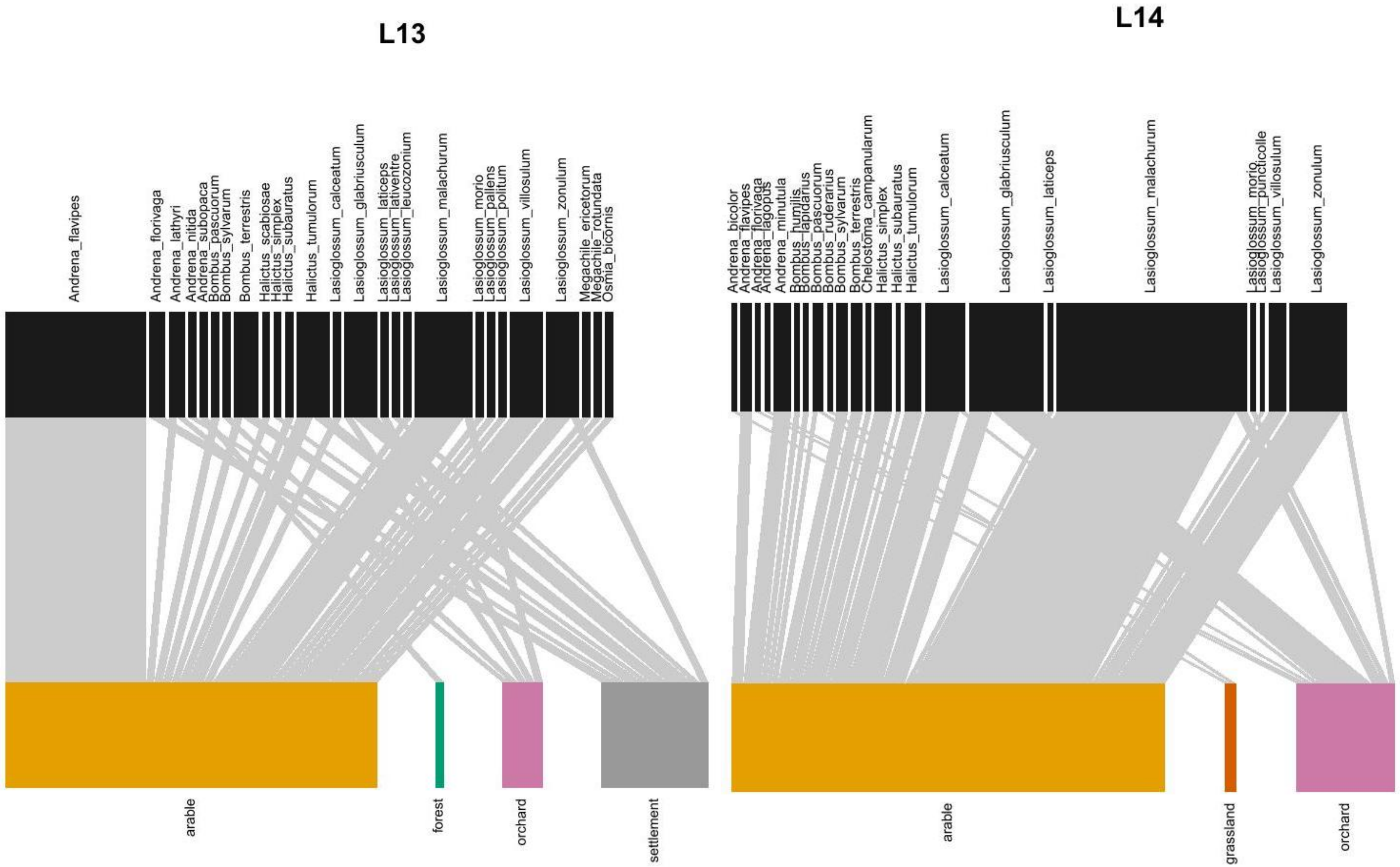

L13
Andrena_flavipes
Andrena_florivaga
Andrena_lathyri
Andrena_nitida
Andrena_subopaca
Bombus_pascuorum
Bombus_sylvarum
Bombus_terrestris
Halictus_scabiosae
Halictus_simplex
Halictus_subauratus
Halictus_tumulorum
Lasioglossum_calceatum
Lasioglossum_glabriusculum
Lasioglossum_laticeps
Lasioglossum_lativentre
Lasioglossum_leucozonium
Lasioglossum_malachurum
Lasioglossum_morio
Lasioglossum_pallens
Lasioglossum_politum
Lasioglossum_villosulum
Lasioglossum_zonulum
Megachile_ericetorum
Megachile_rotundata
Osmia_bicornis
arable
forest
orchard
settlement
L14
Andrena_bicolor
Andrena_flavipes
Andrena_florivaga
Andrena_lagopus
Andrena_minutula
Bombus_humilis
Bombus_lapidarius
Bombus_pascuorum
Bombus_ruderarius
Bombus_sylvarum
Bombus_terrestris
Chelostoma_campanularum
Halictus_simplex
Halictus_subauratus
Halictus_tumulorum
Lasioglossum_calceatum
Lasioglossum_glabriusculum
Lasioglossum_laticeps
Lasioglossum_malachurum
Lasioglossum_morio
Lasioglossum_puncticolle
Lasioglossum_villosulum
Lasioglossum_zonulum
arable
grassland
orchard

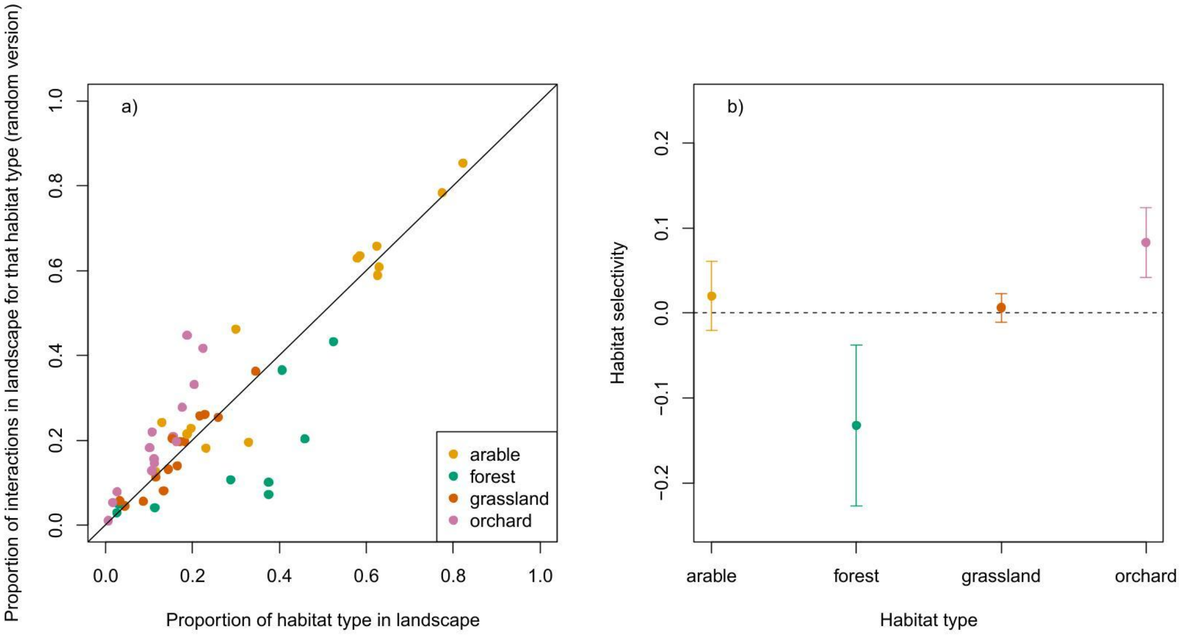

Figure S5: Habitat preference and avoidance of wild bee individuals for the four focal habitat types arable (yellow), forest (green), grassland (brown), orchard (purple). a) For each landscape-habitat combination, proportions of interactions (i.e. proportional wild bee abundance per habitat type) in the respective landscape's species-habitat networks are shown compared to the habitat type's actual proportion in the landscape. Please note that these values are based on probabilistic networks. The diagonal line represents a proportional increase of interactions and habitat proportions. The top left side indicates a wild bee's preference for the habitat in the respective landscape, the lower right side indicates an avoidance. b) Boxplots indicate the preference / avoidance of wild bees (mean and 95% confidence interval) for the focal habitat types arable land (yellow), forest (green), grassland (orange), orchard (purple), summarized for all landscapes.

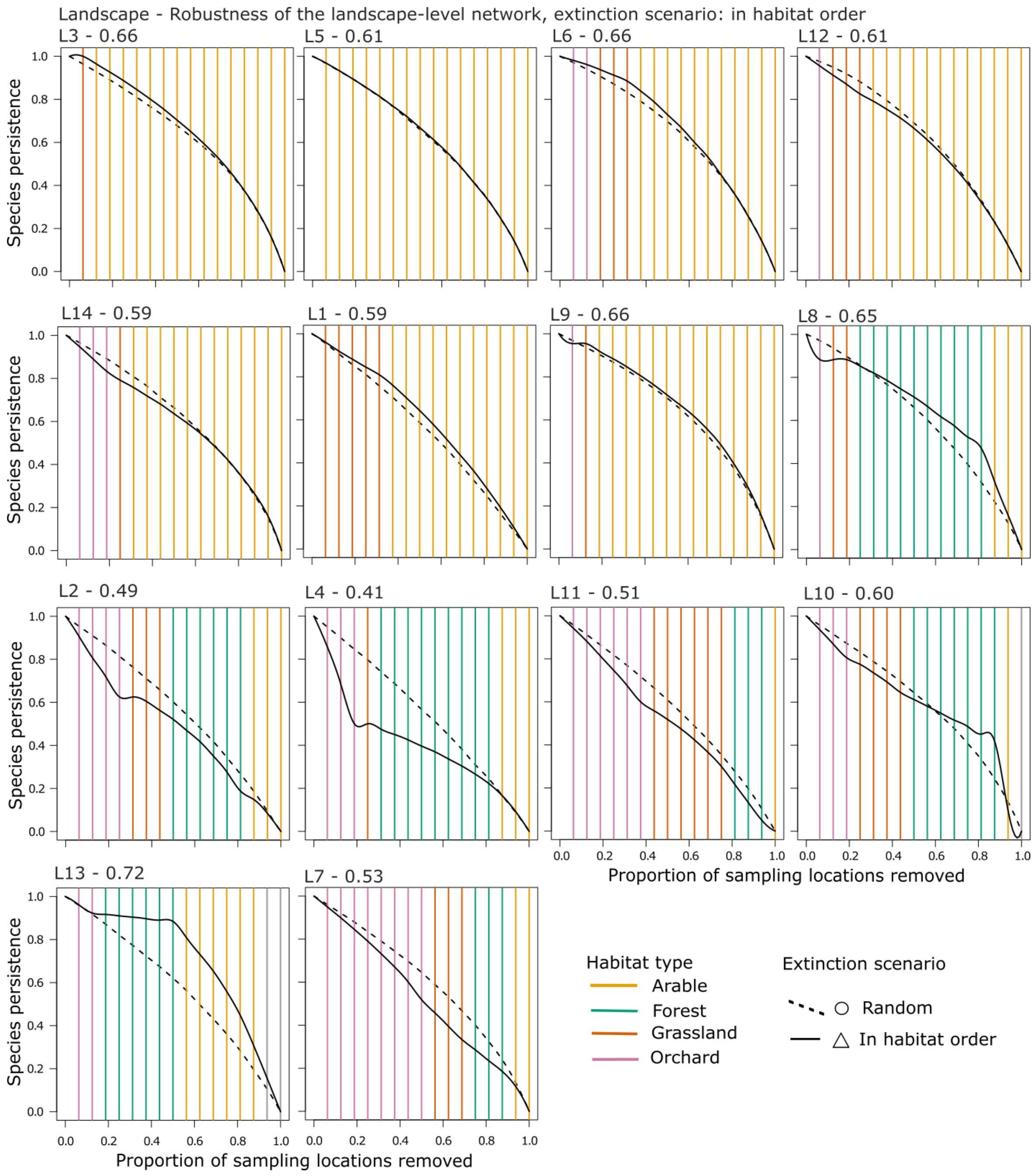

Figure S6: Robustness analysis of all 14 landscapes and their habitat types, sorted by increasing landscape diversity. Species persistence with each extinction step losing a sampling location in the species-habitat network per landscape. Vertical lines stand for the associated habitat type per extinction step for the extinction scenario in habitat order (arable in yellow, forest in green, grassland in brown, orchard in purple). The solid extinction curves indicate the proportion of species being lost at each extinction step when the habitats die out in the order of orchard, grassland, forest, arable. The dashed lines represent completely randomized extinctions (both extinction curves are based on means after 1000 repetitions). Details above the plots indicate the respective landscape ID and the robustness of the network for the extinction scenario in habitat order.

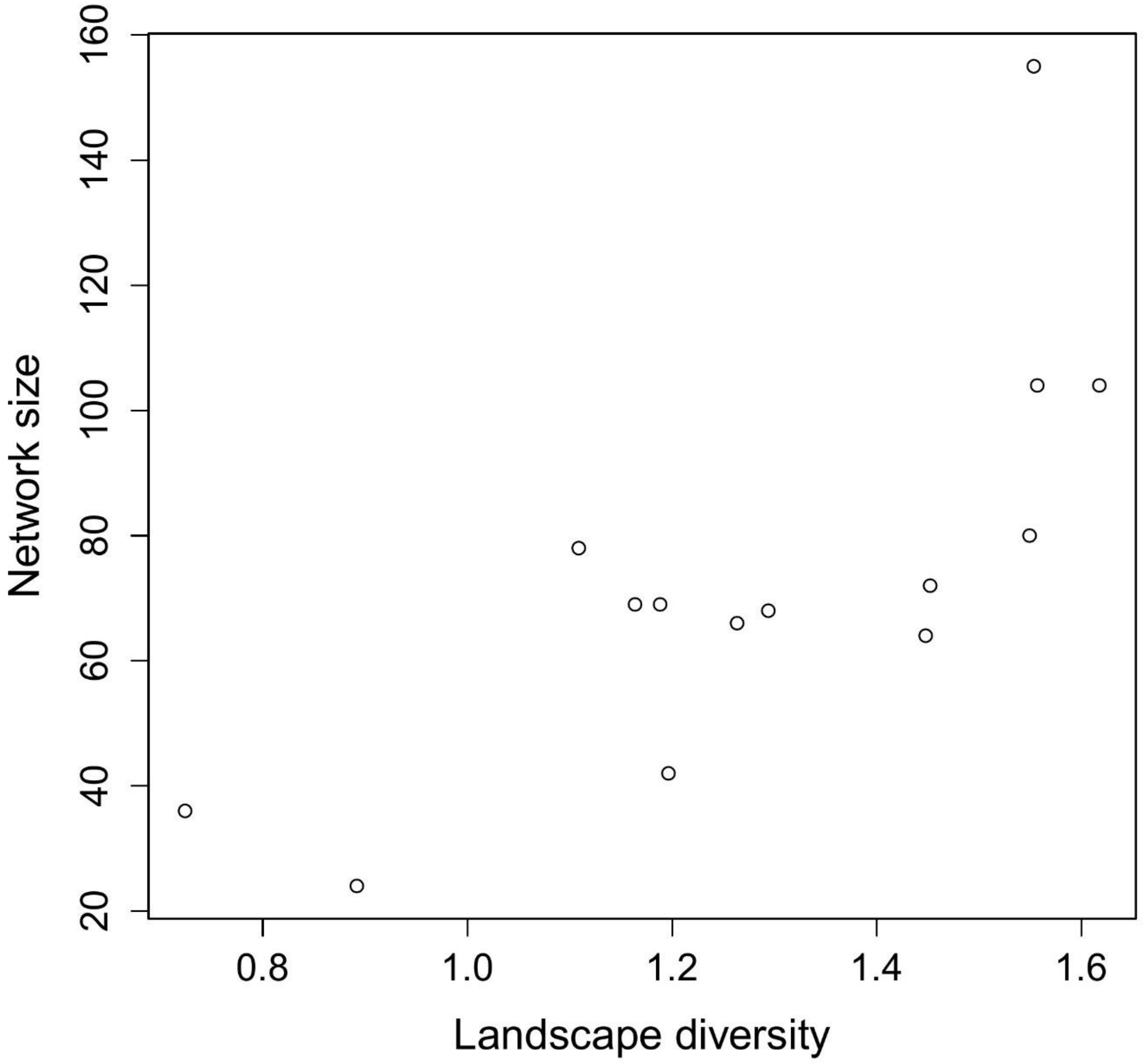

Figure S7: Size of the species-habitat networks for landscapes of differing diversity. Network size is defined as the number of bee species richness (number of columns in network matrix) multiplied by the number of habitat types involved (number of rows in network matrix). Network size is correlated with landscape diversity (Pearson's r = 0.751, P = 0.002).

Section S1: Floral composition

At the time of bee sampling, and in both sampling periods, we assessed the composition of all flowering plant species surrounding each sampling location. For this, we identified the habitat types in a 10 m radius of each pan trap, using the same classification as on the landscape level. Next, we selected ten sampling squares of 1m^2 each within the 10 m radius. The sampling squares were set up randomly in space, but proportionally to the amount of the respective habitat types. For example, habitat proportions of 40% arable land and 60% grassland in the 10 m radius resulted in four squares being put on arable land and the remaining six on grassland. In each sampling square, we identified all herbaceous flowering plants to species level, and identified the floral abundance of each species by counting its floral units. Hereby, a floral unit was defined as a unit "that a medium-sized bee has to fly, rather than walk, between" (Dicks et al., 2002).

Table S1. Overview of floral resources in the four habitat types. These refer to the floral richness and floral units of herbal plants per square meter in 10 m radius around the sampling location. Values refer to means, with minimum and maximum values given in brackets.

	Arable	Forest	Grassland	Orchard
Floral richness per square meter: herbs (in 10m radius)	0.364 (0, 6)	0.103 (0, 4)	0.856 (0, 7)	1.166 (0, 7)
Floral units per square meter: herbs (in 10m radius)	8.418 (0, 400)	2.089 (0, 225)	26.842 (0, 9440)	29.838 (0, 611)

References

Dicks, L.V., Corbet, S.A., Pywell, R., 2002. Compartmentalization in plant-insect flower visitor webs. Journal of Animal Ecology 71, 32–43.

Scherber, C., Beduschi, T., Tscharntke, T., 2019. Novel approaches to sampling pollinators in whole landscapes: a lesson for landscape-wide biodiversity monitoring. Landscape Ecol 34 (5), 1057–1067. 10.1007/s10980-018-0757-2.
